# Evaluating Methods for Isolating Total RNA and Predicting the Success of Sequencing Phylogenetically Diverse Plant Transcriptomes

**DOI:** 10.1371/journal.pone.0050226

**Published:** 2012-11-21

**Authors:** Marc T. J. Johnson, Eric J. Carpenter, Zhijian Tian, Richard Bruskiewich, Jason N. Burris, Charlotte T. Carrigan, Mark W. Chase, Neil D. Clarke, Sarah Covshoff, Claude W. dePamphilis, Patrick P. Edger, Falicia Goh, Sean Graham, Stephan Greiner, Julian M. Hibberd, Ingrid Jordon-Thaden, Toni M. Kutchan, James Leebens-Mack, Michael Melkonian, Nicholas Miles, Henrietta Myburg, Jordan Patterson, J. Chris Pires, Paula Ralph, Megan Rolf, Rowan F. Sage, Douglas Soltis, Pamela Soltis, Dennis Stevenson, C. Neal Stewart, Barbara Surek, Christina J. M. Thomsen, Juan Carlos Villarreal, Xiaolei Wu, Yong Zhang, Michael K. Deyholos, Gane Ka-Shu Wong

**Affiliations:** 1 Department of Biology, University of Toronto at Mississauga, Mississauga, Ontario, Canada; 2 Department of Biological Sciences, University of Alberta, Edmonton, Alberta, Canada; 3 BGI-Shenzhen, Bei Shan Industrial Zone, Yantian District, Shenzhen, China; 4 International Rice Research Institute, Metro Manila, Philippines; 5 Department of Plant Sciences, University of Tennessee, Knoxville, Tennessee, United States of America; 6 Department of Plant Biology, University of Georgia, Athens, Georgia, United States of America; 7 Jodrell Laboratory, Royal Botanic Gardens, Kew, Richmond, Surrey, United Kingdom; 8 Genome Institute of Singapore, Singapore, Singapore; 9 Department of Plant Sciences, University of Cambridge, Cambridge, United Kingdom; 10 Department of Biology and Intercollege Graduate Program in Plant Biology, Huck Institutes of the Life Sciences, Pennsylvania State University, University Park, Pennsylvania, United States of America; 11 Division of Biological Sciences, University of Missouri, Columbia, Missouri, United States of America; 12 Department of Botany and UBC Botanical Garden, University of British Columbia, Vancouver, British Columbia, Canada; 13 Max Planck Institute for Molecular Plant Physiology, Wissenschaftspark Golm, Am Mühlenberg 1, Potsdam-Golm, Germany; 14 Department of Biology, University of Florida, Gainesville, Florida, United States of America; 15 Donald Danforth Plant Science Center, St. Louis, Missouri, United States of America; 16 Department of Botany, Cologne Biocenter, University of Cologne, Cologne, Germany; 17 Department of Plant Biology, North Carolina State University, Raleigh, North Carolina, United States of America; 18 Department of Ecology and Evolutionary Biology, University of Toronto, Toronto, Ontario, Canada; 19 Florida Museum of Natural History, University of Florida, Gainesville, Florida, United States of America; 20 New York Botanical Garden, Bronx, New York, United States of America; 21 Department of Ecology and Evolutionary Biology, University of Connecticut, Storrs, Connecticut, United States of America; 22 Department of Medicine, University of Alberta, Edmonton, Alberta, Canada; University of Glasgow, United Kingdom

## Abstract

Next-generation sequencing plays a central role in the characterization and quantification of transcriptomes. Although numerous metrics are purported to quantify the quality of RNA, there have been no large-scale empirical evaluations of the major determinants of sequencing success. We used a combination of existing and newly developed methods to isolate total RNA from 1115 samples from 695 plant species in 324 families, which represents >900 million years of phylogenetic diversity from green algae through flowering plants, including many plants of economic importance. We then sequenced 629 of these samples on Illumina GAIIx and HiSeq platforms and performed a large comparative analysis to identify predictors of RNA quality and the diversity of putative genes (scaffolds) expressed within samples. Tissue types (e.g., leaf vs. flower) varied in RNA quality, sequencing depth and the number of scaffolds. Tissue age also influenced RNA quality but not the number of scaffolds ≥1000 bp. Overall, 36% of the variation in the number of scaffolds was explained by metrics of RNA integrity (RIN score), RNA purity (OD 260/230), sequencing platform (GAIIx vs HiSeq) and the amount of total RNA used for sequencing. However, our results show that the most commonly used measures of RNA quality (e.g., RIN) are weak predictors of the number of scaffolds because Illumina sequencing is robust to variation in RNA quality. These results provide novel insight into the methods that are most important in isolating high quality RNA for sequencing and assembling plant transcriptomes. The methods and recommendations provided here could increase the efficiency and decrease the cost of RNA sequencing for individual labs and genome centers.

## Introduction

Next-generation sequencing (NGS) has rapidly transformed the life sciences as it is now possible to sequence entire genomes and virtually all expressed genes in a fast and cost-effective manner [1]–[6]. In the case of plant biology, this has made it possible to accelerate fundamental and applied research on how genes interact to form genetic networks [7], the identity of enzymes involved in the biosynthesis of medicinally important primary and secondary metabolites [8], and responses of plants to biotic and abiotic environmental stress [9]. Moreover, NGS is producing massive data sets to answer questions relating to phylogenomic analyses of plant evolution and diversification [10], which is important for extending fundamental results from model organisms to other plant groups, including many that are economically important. Although these exciting developments are quickly changing research across the life sciences, they also present biologists with numerous logistical challenges [6], [11]. Here we describe the development and evaluation of some of the methods and initial results from the One Thousand Plants Consortium (1KP; www.onekp.com), which seeks to provide state-of-the-art molecular tools for hundreds of non-model plant species by sequencing transcriptomes across the diversity of green plants and use these transcriptomes to answer some of the most pressing problems in plant biology [12].

Sequencing plant transcriptomes by NGS is complicated by many factors. The first challenge is to isolate RNA of sufficient quality (i.e., non-degraded RNA, free of impurities) and yield. RNases are particularly problematic in this regard because they rapidly degrade RNA and are widespread in nature and laboratories [13]. A further complication is the myriad primary and secondary plant metabolites (e.g., phenolics, polysaccharides) that vary dramatically within and between species [14]–[17], and can interfere with RNA isolation [18]. A second challenge relates to identifying metrics of RNA quality that predict sequencing success [19], quantified here as the number of long scaffolds (≥1000 bp) assembled from Illumina sequence reads, which provides an estimate of the number of full-length transcripts in a sample. Most genomic sequencing facilities attempt to simultaneously maximize multiple metrics of RNA quality [20], but we are not aware of any large-scale tests that empirically evaluate how these measures affect RNA sequencing (RNA-seq) and downstream assembly of short sequence reads into putatively expressed genes. Finally, the rapidly changing methods associated with sequencing platforms and chemistry will likely have large, but as of yet, unquantified effects on sequencing success [21], [22]. Successful application of NGS technologies to biological problems requires that we understand the impacts of these issues [6].

1KP is seeking to address these problems by sequencing transcriptomes from more than 1000 plant species. The project involves an unprecedented interdisciplinary collaboration of over one hundred researchers from around the world. The overall goal of the project is to create genomic resources for one thousand plant species sampled from across the plant kingdom, including plant species of importance in medicine, agriculture, forestry and conservation. The vast majority of these species are non-model organisms with little to no existing molecular tools. Thus, these data will constitute a cornucopia of genetic information to be used for the biotechnological engineering of crops, development of biofuels, genetic and biochemical characterization of medicinally important plants, and a comprehensive phylogenetic understanding of how plant life has evolved during the past 1 billion years (see www.onekp.com). To achieve 1KP’s goals we have overcome biological, logistical and technological impediments that had hitherto not been attempted on such a scale.

In this paper, we develop and evaluate methods for isolating high-quality total RNA from non-model plants, which can then be used to sequence the diversity of genes expressed within any plant tissue using next-generation sequencing methods. It was necessary for the 1KP project to develop methods that could be employed for the wide phylogenetic diversity of plant species found in nature, from green algae to angiosperms, which diverged >900 mya [23]. To achieve this objective we developed and/or implemented 18 distinct protocols to isolate total RNA from samples; our results and experience showed that no one protocol was suitable for all tissues and species. Using these protocols we sought to answer three questions: 1) what is the success rate of a commonly employed commercial kit for the extraction of total RNA from plants, and which alternative extraction protocols increase the success of isolating RNA; 2) are different plant tissues associated with differences in the quality of total RNA and the size of assembled transcriptomes; and 3) what measures of RNA quality provide the best predictor of the size of assembled transcriptomes into scaffolds?

## Materials and Methods

### Plant Samples and Tissues

We isolated total RNA from 1115 samples from 695 plant species representing 324 families collected from the field, botanical gardens, greenhouses, growth chambers and axenic cultures. No specific permits were required for the collection of samples as the minority of samples collected directly from the field were taken from public land. None of these samples represent endangered or protected species. Samples included non-vascular plants such as algae, hornworts and mosses, and vascular plants including lycopods, ferns, gymnosperms and angiosperms. We isolated total RNA from tissues categorized into one of eight tissue types, including: i) leaf (489 samples), ii) flower (4), iii) fruit (10), iv) buds (leaf or flower) (15), v) shoot/stem (7), vi) below-ground (12; 10 roots, 2 bulbs), vii) mixed tissues (two or more of tissues i–vi) (276) and viii) algal cells (274). Care was taken to properly differentiate and categorize tissue types, but some samples inevitably had overlapping cell types with other tissue types and we therefore view differences among tissues as conservative patterns. For a subset of 71 species, we also tested the effects of tissue age on RNA quality and sequencing success by comparing “young” freshly expanding leaves and “mature” fully expanded but non-senescing leaves collected from tissue that was pooled from at least two healthy plants grown together in the greenhouse. For these samples we used approximately 0.1–0.5 g of tissue from young leaves and roughly 2× as much (up to 1 g) for old leaves; more tissue was required from mature leaves to achieve equivalent concentrations as young leaves (see Results). The complete data set, including the list of all samples, tissues and data, is provided in Table S1.

### Protocols for Isolating Total RNA

No single protocol was optimal for isolating total RNA from all tissues and species. We therefore used 18 distinct protocols to isolate total RNA from samples, where the method used for specific species and tissues was shaped by the experience of individual researchers. Protocols involved commercially available kits (e.g., Qiagen’s RNeasy Plant Minikit), non-commercial RNA extraction lab protocols (e.g., CTAB, acid phenol) and hybrid methods that combined components of both commercial kits and lab protocols. The detailed protocols used for all isolations are available online in Appendix S1.

For all isolations, tissues were collected and flash frozen in liquid nitrogen as soon after collection as possible. In most cases, flash freezing was performed immediately, although in some field collections there was a short delay before freezing, which might account for cases of severe degradation. Slightly less than half of the samples (493) were sent as frozen tissue on dry ice to BGI-Shenzhen (hereafter ‘BGI’), China, for RNA isolation. The remaining samples were isolated by individual labs and sent to BGI as either frozen RNA extracts on dry ice or as dehydrated total RNAs shipped using Genvault’s GenTegra RNA kit (IntegenX, Pleasanton, CA, USA). A comparison of a subset of our samples (N = 168) allowed us to compare effects of shipping liquid RNA versus Genvault dehydrated RNA (see Results).

In answering our first research question relating to the success rates of Qiagen’s RNeasy Plant Minikit and alternative protocols, we deemed samples acceptable for sequencing if they met the following criteria: total RNA concentration ≥150 ng/µl (eluted in 60 µl of RNase free water; note this elution volume only applies to samples shown in Table S2), total RNA mass ≥20 µg, r26S/18S ≥1, RIN ≥8, OD 260/280≥1.9 and OD 260/230≥1.5. We did relax these rigid criteria for samples not shown in Table S2 by sequencing samples of lower quality. By including lower quality samples that did not meet the criteria identified above, this also allowed us to provide a stronger and more comprehensive test of how RNA quality correlated with sequencing success.

### Measures of RNA Quality and Yield

To ensure consistency in measurement of RNA quality, all measurements were performed at BGI. This was especially important because measures of RNA quality outside BGI often deviated substantially from measurements taken at BGI on the same samples, suggesting that methodological differences and degradation during shipping can be important sources of variation in measures of RNA quality. RNA purity was determined by assaying 1 µl of total RNA extract on a NanoDrop 1000 or NanoDrop 8000 spectrophotometer (Thermo Scientific, Wilmington, DE, USA). We measured the optical density (OD) ratio between 260 nm and 280 nm from 526 samples, where pure RNA eluted in H_2_0 (pH 7.0–8.5) or TE (pH 8.0) is expected to exhibit a ratio of 2.0–2.1; deviations below 2 indicate acidic pH or contamination by proteins, phenol and other impurities [13-Appendix 8,24]. We also measured OD 260 nm/230 nm from 406 samples, where pure RNA gives a ratio close to 2. Deviations of OD 260/230 below 2 indicate contamination by polysaccharides, phenol, TRIzol or low pH, which absorb light around 230 nm [18], [24], [25].

**Figure 1:**
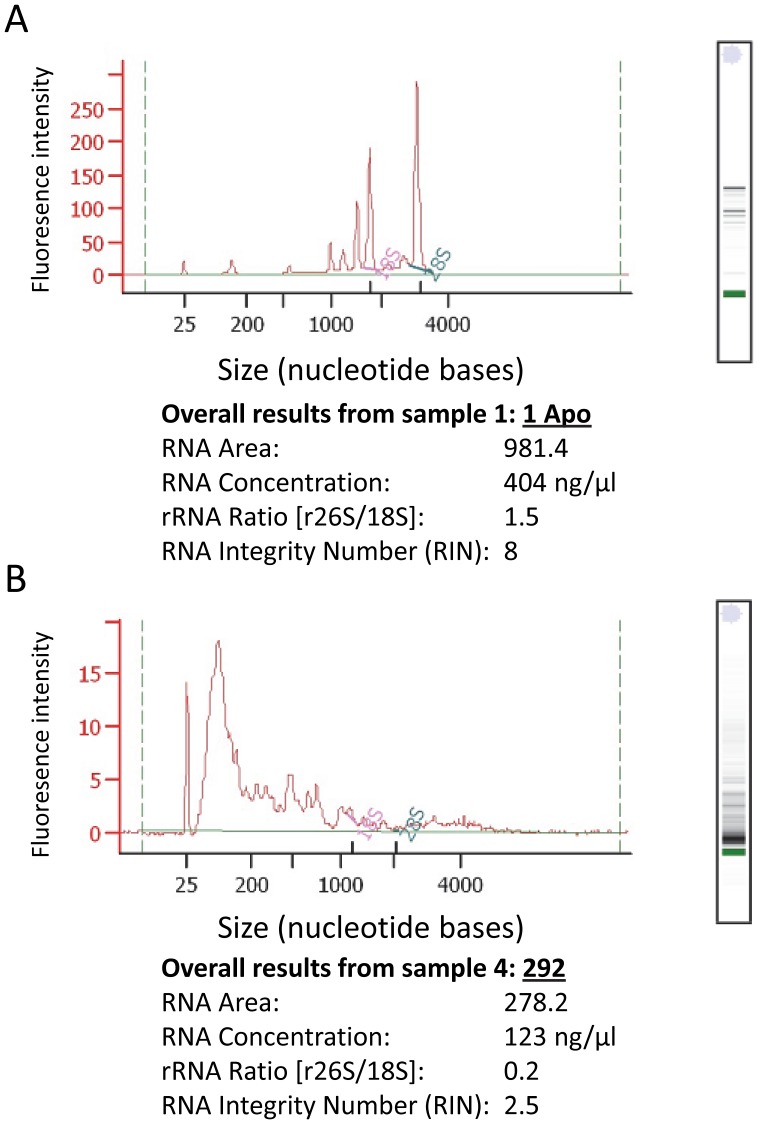
Example of the qualitative and quantitative results from Agilent’s Bioanalyzer 2100. In panels (A) and (B) we show peaks produced from electropherograms (top left) that depict the size distribution of RNA fragments, the corresponding gel-like image of RNA fragments (top right), and metrics of RNA concentration and integrity (r26S/18S and RIN). We show two representative samples with (A) high-quality RNA in terms of high yield and minimal degradation (r26S/18S ≥1, RIN ≥8), and (B) low-quality RNA in terms of modest yield and high degradation (r26S/18S <1, RIN <5). Absence of clear peaks at 26S and 18S and an abundance of short fragments clustering on the left of the electropherogram in panel (B) are the hallmarks of severe RNA degradation. In electropherograms, ribosomal 26S (large) and 18S (small) subunits are shown in green and pink, respectively. Concentrations of 26S and 18S were calculated by taking the area above the green and pink straight lines at the base of the 26S and 18S peaks, respectively.

The concentration, integrity and yield of total RNA were determined by assaying 1 µl of diluted total RNA using Agilent’s 2100 Bioanalyzer with the Plant RNA Nano or Pico chip assay in accordance with the manufacturer’s instructions (Agilent Technologies, Santa Clara, CA, USA). The Bioanalyzer uses electrophoretic technology on a chip to separate RNA fragments by size, which are read by laser induced fluorescence and translated into gel-like bands and peaks (electropherograms) showing the distribution and relative amounts of RNA of different sizes (Figure 1). Concentration (ng/µl) of RNA was determined by comparing the sample with a standard. We measured RNA integrity using two metrics: the ratio of the large (26S) to small (18S) ribosomal RNA subunits (26S/18S) and the RNA integrity number (RIN) [20]. In non-degraded RNA, the quantity of 26S RNA should be 1.6–2× that of 18S, whereas degradation of 26S occurs faster than 18S and thus deviations below the expected ratio are used as a measure of RNA degradation [20]. The use of r26S/18S has been criticized as it might not reflect degradation of other types of RNA such as mRNA [20], [26]–the focus of transcriptome sequencing. RIN has been proposed as an alternative where its calculation is based on a regression model that estimates the integrity of the entire RNA profile, including r26S/18S and the presence and absence of other electropherogram peaks, some of which correspond to mRNA [20]. RIN was originally developed to evaluate the integrity of RNA extracted from mammalian tissues, but plants exhibit greater heterogeneity in ribosomal subunits than animal tissues due to the presence of plastid ribosomes. We therefore selected the plant RNA test program option available in version B.02.07 of the Bioanalyzer software [27], which accounts for the greater heterogeneity of RNA expected from plants.

### Library Construction and Sequencing Methodology

Our study sought to isolate and sequence mRNA from all cells using Illumina’s GAIIx and HiSeq sequencing platforms. Our standard procedure was to isolate polyA RNA from 20 µg of total RNA treated by DNaseI (NEB, Ipswich, MA, USA) using Dynabeads mRNA purification kit (Life Technologies, Grand Island, NY, USA). We used up to 50 µg when the use of a lower mass (typically 20 µg) was insufficient for successful library construction, which we assessed by running final PCR products on an agarose gel; the library construction was considered to have failed when there was no visible band. We used less than 20 µg of total RNA when isolation of an important sample yielded low RNA mass but library construction was successful. Purified polyA RNA was fragmented in a fragmentation buffer (Life Technologies, Grand Island, NY) at 70°C for 90 seconds to 200–300 nt fragment sizes. The first cDNA strand was synthesized with random hexamer primers using the SuperScript II reverse transcription kit (Life Technologies, Grand Island, NY). The second-strand synthesis was performed by incubation with RNase H (Life Technologies, Grand Island, NY) and DNA polymerase (Enzymatics, Beverly, MA, USA). Short double-stranded cDNA fragments were purified using one of two methods. Our standard procedure was to use the QIAquick PCR purification kit (Qiagen, Valencia, CA, USA), whereas for samples with low RNA mass we used Agencourt AMPure beads (Beckman Coulter, Beverly, MA). Both methods were followed by end-repair with Klenow polymerase, T4 DNA polymerase and T4 polynucleotide kinase (Enzymatics, Beverly, MA, USA). A single 3′ adenosine (A base) was added to the double-stranded cDNA using Klenow (3′ to 5′ exo-) (Enzymatics, Beverly, MA, USA) and dATP (GE Healthcare, Buckinghamshire, UK). The Illumina PE Adapter oligo mix was ligated onto the A base on repaired double-stranded cDNA ends and DNA fragments of a selected size were gel-purified to make sure the insert size was 200 bp (±10% deviation). Thereafter, libraries were amplified by 15 cycles of PCR with Phusion DNA polymerase (NEB, Ipswich, MA, USA) and “indexed” paired-end PCR primers; the prepared libraries were 322 bp long. The amplified libraries were denatured with sodium hydroxide and diluted to 2.5 pM in hybridization buffer for loading onto a lane of an Illumina GAIIx or HiSeq flowcell. Read length on the GAIIx platform was typically adjusted to 73–75 bp (mean = 74 bp), but four samples were read at 100 bp. Read length on the HiSeq platform was predominately 90 bp with a small number of sequences with 84–87 bp. All samples were sequenced as paired-end reads, and up to eleven samples were multiplexed into a single lane of the Illumina flow cell.

Prior to assembly of sequences into scaffolds we filtered sequence reads based on four criteria. First, all samples were indexed with unique 6–7 bp sequences and we retained reads with 0 or 1 bp sequence mismatch (>99.5% of reads). Second, we checked for the adapter sequences in the paired sequence reads and rejected pairs with more than 15 bp of continuous adapter sequence, allowing a maximum of three mismatches; this resulted in rejection of ca. 3% of reads. Third, we removed all sequence read pairs where either read had >5% uncalled bases (N’s) (<0.5% of reads). Similarly, we removed sequence read pairs when either read had more than 20 bp with Phred-type Q-score<10 (3.5% of reads). Together these four criteria ensured that contaminant sequences were eliminated from our data and only the highest quality reads were used in assemblies.

We extracted several response variables for analyses from the sequencing results to depict sequencing depth. Specifically, we used total number of bases sequenced, which is the number of bases that exceeded the Phred quality 20 threshold (hereafter “Q20 bases”, a measure of the quality/reliability of sequenced base calls) and the total number of sequence reads.

### Transcriptome Assembly

**Figure 2:**
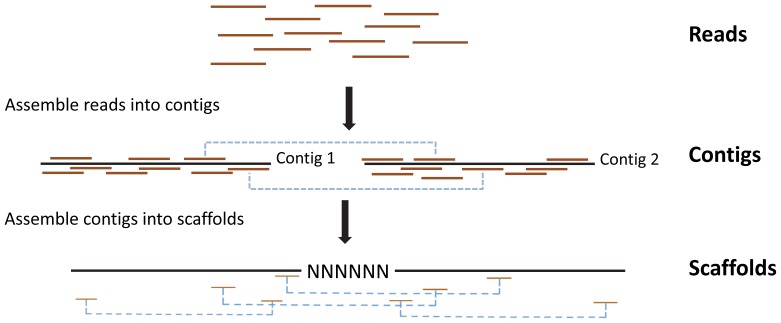
Schematic representation of the method used to assemble Illumina reads into contigs, and contigs into scaffolds. All reads were initially assembled into contigs using the de Bruijn graph method without using information about paired-end reads (shown by blue dashed lines). A contig’s sequence was resolved at every base. Contigs were then assembled into longer scaffolds by connecting contigs that contained paired-end reads assembled into separate contigs. Assembling scaffolds in this way allowed us to create longer sequences of known length, but sometimes there were gaps of unknown sequence. These gaps were constrained to represent <5% of total sequence length.

Transcriptomes were assembled using SOAPdenovo [20], [25], [26], which is designed to rapidly assemble large genomes sequenced on short-read sequencing platforms such as Illumina (Figure 2). When 1KP began in early 2009, this was the best option available. Over the last year, other assemblers (e.g., Trinity, Oases, TransABySS) [6], [27], [28] have been introduced that are specifically designed for assembling transcriptomes, where coverage varies from gene to gene because of differences in expression and alternative splicing. We have repeated the assemblies using a new software SOAPdenovo-trans (http://soap.genomics.org.cn/SOAPdenovo-Trans.html) that is faster than Trinity and recovers more full-length transcripts from the same data set, based on a comparison to the annotated genomes for rice and mouse (unpublished results). We find that the number of large (≥1000 bp) scaffolds from our original assemblies and the newer assemblies are highly correlated (*r*_Pearson_ = 0.77, *P*<0.001, *N* = 626), indicating that the original assemblies capture a large amount of the variation explained by higher yielding newer assemblies. We therefore decided to base our analyses on the older assemblies, since they more honestly reflect the basis on which project decisions were made and SOAPdenovo-trans has not yet been formally published, although it is available to the public (see above). Moreover, a comparison of large scaffolds (≥1000 bp) from our SOAPdenovo assemblies to GenBank’s non-redundant protein sequence database (nr) using the BLASTx sequence translation tool containing all vascular plants and algae showed that our assemblies reliably assembled known proteins; on average 88% ±1% (95% CI; median = 95%) of scaffolds matched a known protein with high confidence (E<10^−10^).

Our assembly methods followed Li et al. [21]. In brief, SOAPdenovo uses the de Bruijn graph method to represent all reads, with a K-mer designated as a node and (K−1) base overlaps between two K-mers as an edge. We used a single K-mer length of 29 for all assemblies because preliminary analyses and other published studies have shown that a single K-mer of length 25–31 can recover a large number of transcripts with variable expression levels [11]. Although adjusting K-mer length or using multiple K-mers can optimize the assembly of individual transcriptomes [28], [29], we used a single K-mer in this study to facilitate comparisons of transcriptome results across all samples based on a standard and consistent method of assembly. The alternative of optimizing samples by K-mer length could increase total number of transcripts identified, but it would have the undesirable effect of confounding our results with differences in methods of assembly; this ad hoc approach was impractical given the number of samples. Some tips and low-coverage K-mers in the graph were removed to eliminate branches and reduce problems induced by sequencing errors. The de Bruijn graph was then converted into a contig graph by turning the series of linearly connected K-mers into precontig nodes using the merging option for the similarity of sequences (–M) equal to 2. Dijkstra’s algorithm detected bubbles, which were then merged into a single path if sequences of branches were sufficiently similar. By this approach, nearly identical sequences could be assembled into consensus “contig” sequences, where every base was defined in an uninterrupted linear sequence. Contigs were then connected by paired-end reads joined by a known distance to form a scaffolding graph (Figure 2). Edges in this graph were connections between contigs, and the edge length was estimated from the insert size (200 bp) of the paired reads. We retained all scaffolds ≥100 bp where ≥95% of bases were defined (Figure 2).

From each assembled transcriptome the response variable was the number of scaffolds ≥1000 bp, which ideally represents an estimate of the number of full length transcripts in a sample. There are potential pitfalls with defining genetic diversity of transcriptomes in this way. For example, if we focus on the number of scaffolds of all lengths as our response variable, then some genes will be represented by many small scaffolds. We clearly see this in our samples because the number of scaffolds ≥100 bp frequently exceeds 100,000, which is much larger than conventional estimates of the number of genes expressed in plant tissues [30]–[34]. By contrast, if we only count scaffolds that meet a large threshold cutoff, then we increase the probability of each scaffold representing a unique full-length transcript, but potentially miss legitimate smaller genes.

We determined the optimal threshold size of scaffolds by evaluating a range of cutoffs. We performed separate analyses on scaffold assemblies with minimum cutoffs set to 500 bp (i.e., all scaffolds were ≥500 bp), 600 bp, 700 bp, 800 bp, 900 bp and 1000 bp. The strength of Pearson product moment correlations (*r*) between data sets that varied in the threshold size of scaffolds ranged between 1.00 (1000 bp vs. 900 bp) and 0.78 (1000 bp vs. 700 bp), with *P<*0.001 for all correlations (Table S3). In other words, a transcriptome data set with a minimum cutoff of 500 bp or 1000 bp contains largely overlapping information about the estimated number of genes expressed in a sample’s transcriptome. We focus here on the number of “large” scaffolds (≥1000 bp, with at least 950 resolved bp) in our results for three reasons: 1) there is a high correlation between the number of large scaffolds (≥1000 bp) and the number of scaffolds assembled using smaller size cutoffs (i.e., 500 bp, 600 bp, 700 bp, 800 bp and 900 bp) (Table S3); 2) large scaffolds explained the greatest variation in most statistical models presented in the results; and 3) large scaffolds exhibited a higher proportion of high confidence matches to known proteins in BLASTx than smaller thresholds (data not shown).

### Statistical Analyses

#### Effects of tissue type

We tested the effect of tissue type on RNA quality, RNA yield, sequencing depth (i.e., number of base pairs sequenced, number of Q20 bases and number of sequence reads), and number of long scaffolds assembled using Proc Mixed in SAS 9.1 (SAS Institute, Cary, NC, USA). We first used a likelihood ratio test (LRT) to determine whether an unequal variance model (i.e., estimating variance separately for each tissue type) provided a better fit for our data than a standard model that assumes homogeneity of variance. We then assessed whether our data met assumptions of normality, and we transformed data accordingly using either log or square root transformations. Pairwise differences between least-squares mean values of tissue types were compared using the Tukey-Kramer adjustment for multiple comparisons implemented using the ADJ option in SAS. Mean values were subsequently back-transformed to their original units for illustrative purposes.

It is widely believed that harvesting the youngest tissue results in higher RNA quality and yield [35, p. 116]. To test this conventional wisdom, we systematically harvested young freshly expanding tissue and fully expanded mature tissue (but not “old” or senescing) from a phylogenetically diverse subset of 71 species as described above (Table S1). The statistical model included tissue age as the main effect with species included as a blocking factor.

#### Predicting the size of assembled transcriptomes

We attempted to understand the factors that provide the best predictors of the size of the assembled transcriptome, measured as the number of large scaffolds (≥1000 bp). We included four types of variables in the analyses explained below: 1) tissue type, 2) measures of RNA quality and yield, 3) factors associated with sequencing methodology, and 4) a covariate for sequencing depth (number of bases). Measures of RNA quality and yield included RNA concentration (ng/µl), total RNA yield or mass (µg), r26S/18S, RIN, OD 260/280 and OD 260/230. Sequencing methodology refers to factors that were under experimenter control; this included the mass of total RNA sequenced (µg) and the sequencing platform (Illumina GAIIx vs. HiSeq), which was also correlated with the length of sequence reads (GAIIx: 74 bp/100 bp, HiSeq: 90 bp).

We achieved on average 2 Gb of data across all samples (with an enforced minimum of 1 Gb and a practical maximum of 4 Gb). Nevertheless, there was variation in the number of bases sequenced (i.e., sequencing depth) and we included this variable as a covariate in analyses.

We performed two sets of analyses because OD ratios were measured for a smaller number of samples than the other variables. In the first analysis, we included all nine variables described above, and we used maximum likelihood to compare the explanatory power of all possible linear combination of factors (511 possible models). The best model was identified according to the lowest Akaike information criterion (AIC) value; AIC measures the explanatory power of the model weighted by the number of parameters included, where lower AIC corresponds to greater explanatory power. As per convention, nested models with AIC values that differed by >2 (ΔAIC) were viewed as significantly different, whereas nested models with ΔAIC <2 were seen as statistically equivalent [36]. Because AIC values are sensitive to variation in sample size, only samples for which we had data on all variables were included in the analysis; the total size of this “balanced” data set was 184 samples. However, all data were used in the calculation of the variation explained by each factor (partial r^2^) because this statistic is less sensitive to variation in sample size. The second analysis used the larger balanced data set of 512 samples, which lacked the OD ratios but contained all other variables. This data set considered all possible combinations of seven explanatory variables (127 models in total) and selected the best model according to the methods described above.

We focused our results on analyses that used scaffolds ≥1000 bp but we also performed analyses on smaller threshold cutoffs. Comparing results of the best-fitting models produced qualitatively identical results for all data sets with respect to the direction and statistical significance of explanatory variables. The only exception to this was that tissue type became a significant predictor of the number of scaffolds when the minimum threshold scaffold size was set to 600 bp, but this result was only found for the large data set without OD ratios.

## Results

### Likelihood of Isolating High-quality RNA

When presented with a previously unstudied plant tissue, most researchers first attempt RNA isolation using one of several commercially available plant RNA isolation kits. To provide researchers with *a priori* guidance on the likelihood of success we asked: What is the success rate of a commonly employed commercial kit in extracting high-quality RNA for NGS (see Appendix S1)? For each of 81 samples that represented a wide phylogenetic diversity of plants we isolated total RNA from fresh leaf material using Qiagen’s RNeasy Plant Minikit (Appendix S1, Protocol 1; Table S2); 46% of these samples met our criteria for yield and quality of total RNA (see Methods). We attempted an additional extraction using RNeasy’s alternative protocol (Appendix S1, Protocol 2) on 37 of the samples that failed using Protocol 1; 32% (12) of these samples met our criteria for RNA quality and mass. Thus, using a commonly used commercial extraction kit (Protocol 1) and its prescribed alternative modification (Protocol 2), we isolated total RNA from 66% of samples that met our criteria for sequencing. However, certain taxa never yielded high-quality RNA using commercial kits, and therefore 16 alternative protocols were developed and/or implemented for the remaining samples (see Appendix S1).

### Effects of Tissue Type on RNA Quality and Assembled Transcriptome Size

**Figure 3:**
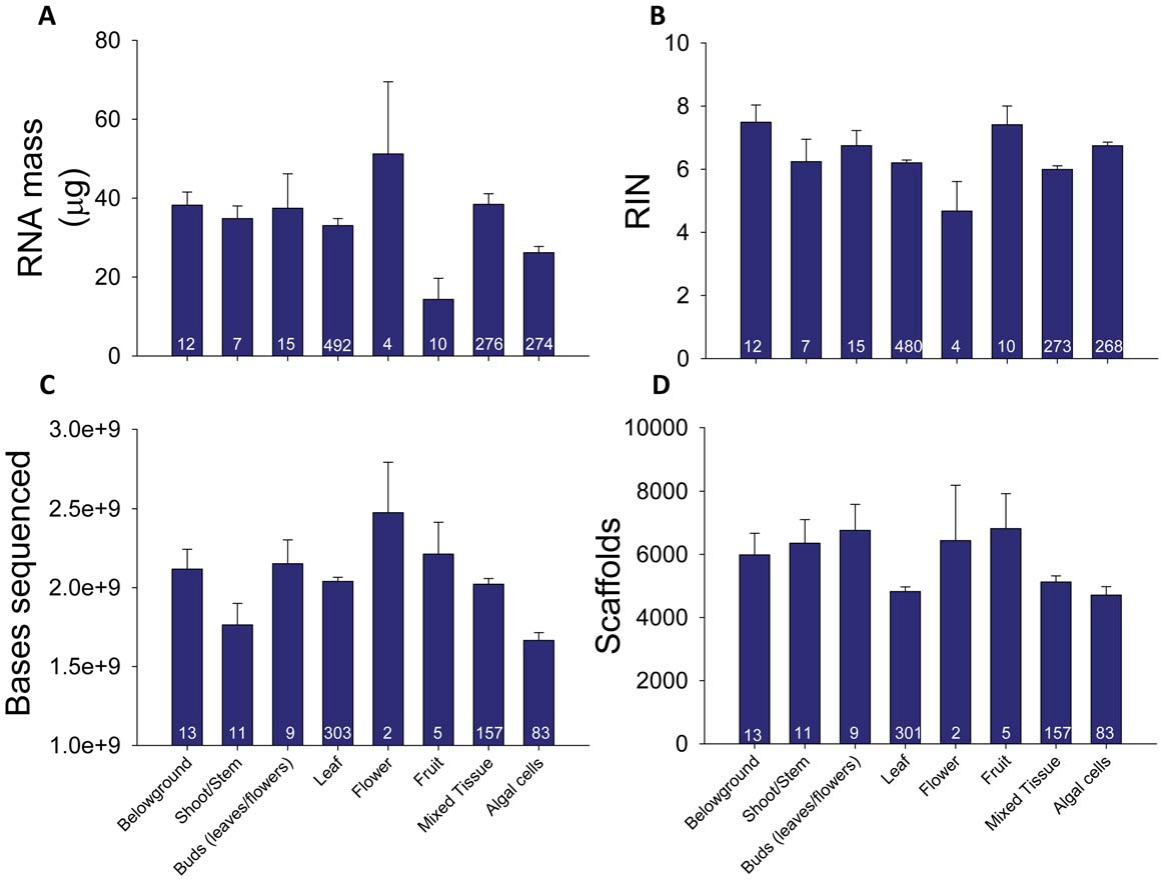
Variation among tissue types in RNA quality and transcriptome size. We observed differences among tissue types for (A) total RNA mass (µg) isolated, (B) RIN, (C) sequencing depth and (D) number of scaffolds. For each tissue, we show the mean +1 SE and sample size at the base of columns. A posteriori pairwise contrasts among means corrected for multiple comparisons are shown in Supplemental Tables 3 and 5.

Yield and quality of total RNA isolated from samples varied substantially among plant tissue types. The mass of extracted RNA varied among tissues by more than 350% (*P = *0.007), with flowers, buds and tissue mixtures yielding the most RNA and fruits producing the least (Figure 3A, Table 1, Table S4, S5). Measures of RNA quality, including r26S/18S, RIN (Figure 3B), and OD 260/280, also significantly varied among plant tissues (Table 1). With the exception of a fairly strong correlation between r26S/18S and RIN (*r* = 0.64, *P*<0.001), descriptors of RNA quality were not highly correlated with one another. For example, although the mass of RNA isolated from flowers was 3.5× greater than RNA mass from fruits (*P = *0.03), average RIN was 37% lower in flowers than in fruits, although not significantly (*P = *0.21). Means and statistical comparisons among all tissues are available online (Table S4, S5).

**Table 1:** Effects of tissue type and age on metrics of RNA quality and sequencing.

	Tissue type							Tissue age				
	ndf^2^, ddf^3^			F^4^		P^5^		ndf, ddf		F		P
**RNA quality**												
RNA mass^1^		7,24	3.77		**0.007**		1,40		1.36		0.25	
r26S/18S		7,1071	10.06		**<0.001**		1,41		4.56		0.33	
RIN		7,1061	5.14		**<0.001**		1,41		48.91		**<0.0001**	
OD 260/280		6,503	3.16		**0.005**		1,40		0.58		0.45	
OD 260/230		6,383	1.30		0.26		–		–		–	
**Sequencing**												
Bases		7,576	7.95		**<0.001**		1,31		2.63		0.12	
Q20 bases		7,576	13.23		**<0.001**		1,31		2.66		0.11	
Reads		7,576	10.98		**<0.001**		1,31		2.54		0.12	
Scaffolds		7,574	2.33		**0.024**		1,31		0.87		0.36	

Significant effects (*P<*0.05) are shown in bold.

1 Measured as µg of total RNA isolated from a given tissue.

2 Numerator degrees of freedom (ndf) of F-statistic.

3 Denominator degrees of freedom (ddf) of F-statistic. ddf are low for RNA mass because an unequal variance model was used to account for heteroscedasticity in residuals among tissues.

4 F-statistic from analysis of variance (ANOVA).

5 P-value of F-statistic given ndf and ddf.

Across all sequenced samples, our assembly of transcriptomes recovered a large number of putative transcripts, with a mean of 15,512 scaffolds ≥500 bp (±192 [stderr], *N* = 629 samples) in length, 7444 scaffolds ≥800 bp (±124 [stderr], *N* = 629) and 4997 scaffolds ≥1000 bp (±99 [stderr], *N* = 629). As described in the methods, 88% (±1%, 95% CI) of long scaffolds (≥1000 bp) matched a known protein in the GenBank nr protein database for algae and plants with high confidence (<E^−10^). All measures of sequencing depth and transcriptome size varied significantly among plant tissues (Table 1). Number of bases sequenced, number of bases sequenced with high-quality (“Q20 bases”), and number of sequence reads were consistently lowest in green algal cells (hereafter just “algae” or “algal cells”) and highest in floral, fruit and bud tissues (Figure 3C, 3D, Table S6). There was weaker but still significant variation among plant tissues for number of large scaffolds (≥1000 bp) (Table 1, Figure 3D). Again, algal cells yielded the lowest number of scaffolds, whereas flowers, fruits and buds returned the highest numbers of scaffolds. However, these last contrasts were never significant in a posteriori pairwise comparisons that corrected for multiple tests (Table S7).

Unexpectedly, age of tissue (i.e., young freshly expanding vs. mature fully expanded but non-senescing tissue) had only a weak effect on RNA quality and no clear effect on sequencing results (Table 1). Although younger leaves gave higher yields of RNA (see Methods), the only effect of tissue age on RNA quality was seen for RIN (*F*_1,41_ = 48.91, *P*<0.001), which was 13% higher in young tissues (mean = 6.93, SE = 0.08) than in mature tissues (mean = 6.14, SE = 0.07). There was no clear effect of tissue age on any metric of transcriptome size including the number of large scaffolds (Table 1). The only exception to this result was seen when we reduced the minimum threshold size cutoff of scaffold assemblies to 500 bp, at which point young tissue produced 7.5% more scaffolds than older tissue (*F*_1,31_ = 7.1, *P = *0.01).

### Predicting Size of Assembled Transcriptomes

**Figure 4:**
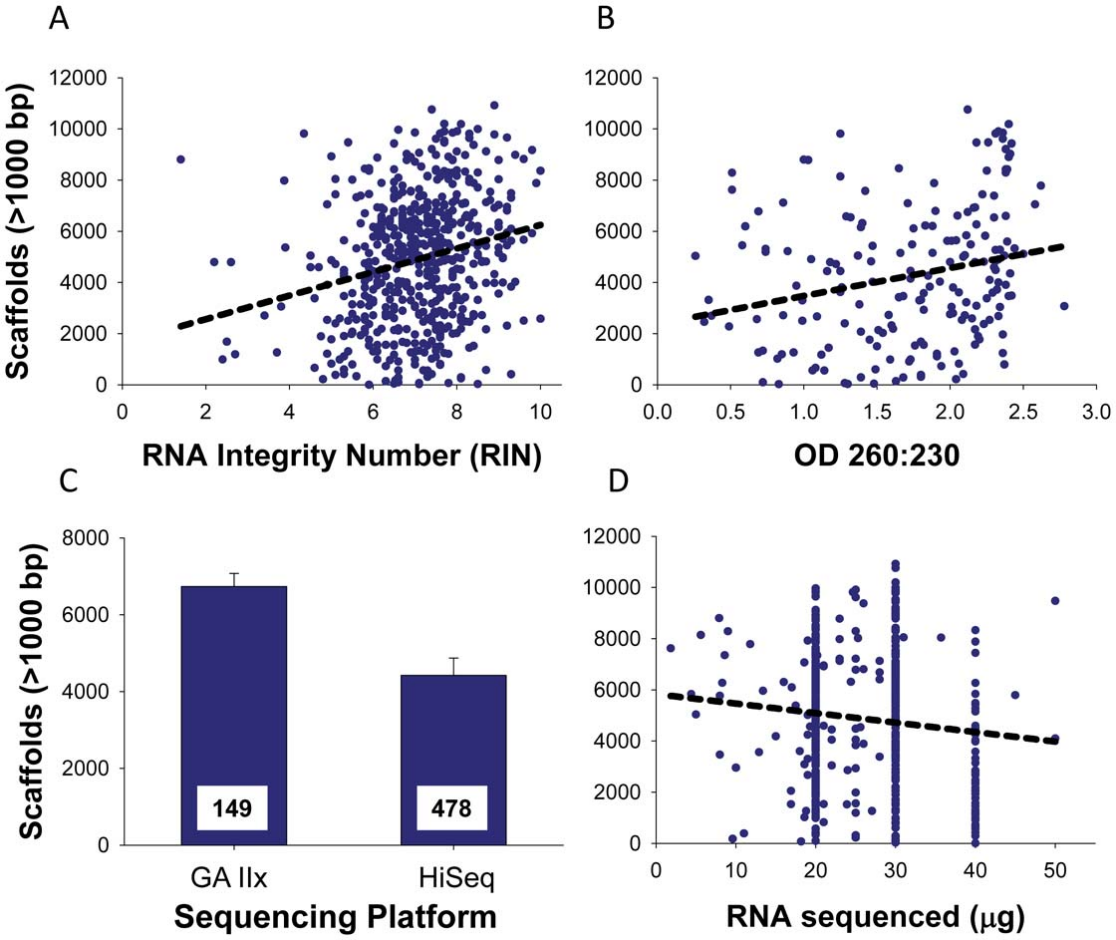
Factors that significantly predicted the number of large scaffolds. Among our measures of RNA quality, (A) RNA integrity number (RIN) and (B) OD 260/230 ratio were the strongest predictors of the number of scaffolds ≥1000 bp. (C) Sequencing platform also had a strong effect on number of large scaffolds (*P*<0.001, Table 2; numbers at the base of bars show sample size), and (D) mass of RNA sequenced had a weak but detectable effect (see Table 2). Note, for most samples we used 20, 30 or 40 µg of total RNA for sequencing, but a few samples used intermediate or lower amounts.

RNA quality and covariates associated with sequencing methodology both predicted the number of large scaffolds assembled from individual samples. For the data set that included OD ratios, we compared all 511 possible linear models (Table S8) using maximum likelihood statistics (see Methods). The best-fitting model (AIC = 3187) contained seven explanatory variables that accounted for 36% of the total variation in the number of scaffolds (Table 2, Table S8). This best-fitting model included tissue type, r26S/18S, RIN, OD 260/280, OD 260/230, as well as sequencing platform (GAIIx vs. HiSeq) and the mass of total RNA used in library construction; a model that also included RNA concentration fit almost equally well. Among variables relating to tissue type and RNA quality, only RIN and OD 260/230 were significant predictors of the number of large scaffolds (Table 2). Both RIN and OD 260/230 were positively correlated with the number of large scaffolds, and together they accounted for 8.9% of the total variation (Figure 4, Table 2). Variables associated with sequencing methodology accounted for most of the variation in the number of large scaffolds. Specifically, there were significantly fewer scaffolds from transcriptomes sequenced on the HiSeq platform compared to sequences generated from the GAIIx platform; this one factor accounted for 22% of the total variation in the number of large scaffolds (Figure 4, Table 2). This result was unexpected because the HiSeq platform had a nominally longer read length (90 bp) than the GAIIx platform (74 bp). There was also a weak yet significant negative relationship between mass of total RNA used to construct libraries for sequencing and number of scaffolds (Table 2).

**Table 2:** Statistical significance of explanatory variables in the best-fitting models for the data set with OD ratios and without OD ratios.

Variable	df^1^	F	P	r^2^
**OD ratios included**				
Tissue type	2,174	0.23	0.791	0.002
Sequencing platform	1,174	59.27	**<0.001**	0.219
r26S/18S	1,174	0.90	0.344	0.003
RIN^2^	1,174	9.38	**0.003**	0.035
RNA seq^3^	1,174	8.11	**0.005**	0.030
OD 260/280	1,174	3.46	0.065	0.013
OD 260/230	1,174	14.69	**<0.001**	0.054
**OD ratios excluded**				
Tissue type	7,499	0.28	0.96	0.003
Sequencing platform	2,499	30.35	**<0.001**	0.104
r26S/18S	1,499	1.00	0.318	0.002
RIN	1,499	8.08	**0.005**	0.014
RNA seq	1,499	12.77	**<0.001**	0.022

The best-fitting models were determined by comparing AIC values among models that considered all possible combinations of explanatory variables. Statistical significance was determined using an ANOVA model with type III sums-of-squares (SS). Variables with *P<*0.05 are shown in bold. Partial r^2^ values (coefficient of determination) were determined by dividing SS values of each factor by total SS.

1 Numerator (first number) and denominator (second number) degrees of freedom (df) for F-test.

2 RNA integrity number (RIN).

3 Mass of total RNA sequenced.

Other abbreviations as per Table 1.

We also used a larger data set (see Methods) that lacked OD ratios to predict the number of large scaffolds. Among all possible linear models (Table S9), the model with the highest information content included the same variables (tissue type, r26S/18S, RIN, sequencing platform and the amount of RNA sequenced; AIC = 9174) as the best-fitting model described above (minus the OD ratios). This model accounted for less total variation (14.5%) in the number of large scaffolds than the previous model (Table 1). As with the smaller data set, RIN, sequencing platform and total amount of RNA sequenced were significant predictors of the number of large scaffolds, and directionality of correlations was identical to those described above (Figure 4). In this larger data set, number of bases sequenced was also positively correlated with number of scaffolds (*P<*0.001, r^2^ = 0.02), but including the number of bases in the model led to a weaker overall fit of the data (ΔAIC = 15.9) compared to models that excluded this covariate.

Finally, we evaluated the optimal method of shipping RNA using a subset of samples (see Methods). Samples were either sent as frozen RNA extract on dry ice (N = 100) or as dehydrated total RNA (N = 68) using Genvault’s GenTegra RNA kit (IntegenX, Pleasanton, CA). Genvault samples had 17% more scaffolds on average (5675±265 scaffolds) than samples sent as frozen extracts (4842±211 scaffolds) (*F*_1,167_ = 6.29, *P = *0.013), and this effect explained 3.6% of the total variation in the number of scaffolds among samples.

## Discussion

Three results from our study have important implications for the isolation, sequencing and assembly of phylogenetically diverse plant transcriptomes. First, we identified specific tissues and metrics of RNA quality that provide significant predictors of the number of large scaffolds, but these variables explained relatively little total variation in the data. Second, components of sequencing methodology (e.g., sequencing platform) had a larger impact on the number of scaffolds than metrics of RNA quality. Third, Illumina sequencing and assembly of scaffolds were fairly robust to variation in RNA quality. Based on these results, we recommend researchers and genomic facilities optimize RNA quality and sequencing methodology as much as possible, but still sequence samples of low to moderate quality as these samples can yield large transcriptomes sequenced by Illumina.

### Effects of Tissue Type on RNA Quality and Transcriptome Sequencing

As expected, quality of RNA and size of transcriptomes varied among tissue types [30], but not always in ways that were consistent with conventional wisdom. Some tissues such as flowers and roots resulted in a high yield and quality of total RNA, whereas the most commonly harvested tissue (leaves) resulted in relatively modest to low RNA yields and quality (Figure 3, Table S4). Although many of these results show strong statistical support, we caution that they should be viewed tentatively given the low sample sizes and limited phylogenetic coverage of some tissues. Despite the large differences in RNA quality and sequencing depth, we observed little variation in the number of scaffolds among tissues (Figure 3D). Leaves did contain fewer scaffolds than other tissues, but no tissues were significantly different from one another in pairwise comparisons that adjusted for type-I error (Table S7). Perhaps the most surprising result was the lack of a large difference in RNA quality between young and mature leaves (Table 1). Many researchers report difficulties in extracting nucleic acids from older tissues [35, p. 116], and our result showing that young tissue has higher RNA integrity and yield compared to mature tissue partially supports this view. Despite this fact, our results also show that if RNA can be extracted, most measures of RNA quality are comparable between young and mature tissues, and the number of large scaffolds is statistically equivalent.

### Predicting Size of Transcriptomes

Our results identify specific metrics of RNA quality that predict the size of assembled transcriptomes. Specifically, measures of RNA integrity (RIN) and purity (OD 260/230) predicted the number of large scaffolds assembled from samples. Since RIN quantifies RNA degradation [20], our results imply that degraded RNA prevents assembly of large scaffolds from sequenced transcriptomes. Similarly, our results indicate that impurities in RNA samples interfere with cDNA synthesis from mRNA because OD 260/230 ratios below 2 indicate the presence of contaminants (e.g., salts and organic compounds). This result is expected given studies that show impurities, including ubiquitous plant secondary metabolites, can inhibit reverse transcriptase [37], [38].

Although we identify significant predictors of transcriptome sequence/assembly quality, it is noteworthy that these predictors account for less than 10% of the total variation in the number of large scaffolds. There are two principal explanations for this weak predictive value. First, transcriptomes are by their very nature complex mixtures of many types of RNA that dynamically change in space and time [30], [32], and we specifically targeted mRNA, which comprises only 1–5% of the total RNA in a cell [13]. As such, all metrics of total RNA quality are bound to be coarse depictions of quality and quantity of mRNA. Second, sequencing and assembling transcriptomes is associated with many steps in which noise and bias can be introduced to obscure an otherwise clear effect of RNA quality [39]. Therefore, our results suggest that maximizing RIN and OD 260/230 will increase success of transcriptome sequencing to a point (Figure 4A, 4B), but it is still possible to sequence and assemble transcriptomes with suboptimal levels of these and other measures of RNA quality.

The most counter-intuitive result was the relationship between sequencing platform and number of large scaffolds. Contrary to our expectation, Illumina’s HiSeq platform, which produced predominantly 90 bp reads, resulted in 34% fewer large scaffolds than samples sequenced on Illumina’s GAIIx platform, which produced 74 bp reads. This result is surprising because the HiSeq platform was designed to increase sequencing depth and produce longer high-quality reads that should facilitate the assembly of longer scaffolds and larger transcriptomes. Why then do we find the opposite to be true in our large and phylogenetically diverse data set? One possibility is that the sequencing chemistries of the HiSeq and GAIIx platforms are biased in their ability to sequence GC-rich templates, which could lead to systematic biases when sequencing inherently GC-rich mRNA. This does not seem to explain our result because a comparison of HiSeq and GAIIx platforms finds no clear bias in %GC content of scaffolds (Figure S1). A second possible explanation is that samples sequenced on the HiSeq platform represent a phylogenetically biased subset of species with a lower diversity of expressed genes, such as algae [40], [41]. This is also an unlikely answer. For example, algae were disproportionately represented on the HiSeq platform (18% of HiSeq samples were algae vs. 7% of GAIIx samples), but numbers of large scaffolds sequenced from algae and non-algae were statistically equivalent (*P = *0.17) when we restrict our analysis to just HiSeq samples.

A third possible explanation relates to differences in the true quality of sequenced bases on the two platforms. Sequence quality is quantified as Q-scores, where higher Q indicates lower sequencing error. Unfortunately, no direct comparison of Q-scores can be made between samples sequenced on the GAIIx and HiSeq platforms because the software used to estimate Q-scores on the GAIIx platform systematically underestimates Q-scores and thus over-estimates error rates [22], whereas recent versions of the software used for the HiSeq platform quantify error rates of sequenced base pairs more accurately [22]. Hence the number of high quality bases that are useful for transcriptome assembly is higher than indicated for the GAIIx platform, even when we compare the two platforms by a simple Q20 threshold, because they do not define Q values in an equivalent sense.

### Practical Advice for Sequencing Plant Transcriptomes

Given the results of this study and the collective experience of the many collaborators involved in 1KP, we are able to provide plant researchers with practical recommendations for maximizing success of isolating high-quality total RNA from plants for NGS (also see Appendix S1). First, flash-freezing fresh tissue immediately upon collection ensures the highest quality RNA with the least degradation [26]. Second, using flower, bud or young root tissues can facilitate the isolation of high-quality total RNA [35, p. 116]. Third, smaller amounts of young tissue generally yield as much RNA as larger amounts of mature tissue. Fourth, use of commercially available kits and easily implemented hybrid protocols are the most efficient method for successfully isolating total RNA for NGS when working with new types of tissue or previously unstudied species. If these extractions do not provide the desired result we recommend choosing the isolation protocol in Appendix S1 used for the taxa most closely related to the focal organism of the experimenter (see Table S1). Fifth, working in an RNase-free environment is essential for successful isolation and preservation of total RNA. Sambrook and Russell [13] provided excellent advice on how to avoid RNase contamination, and we emphasize here that various chemicals can be added to solutions (e.g., DEPC-treated water) as well as to working surfaces and equipment (e.g., RNase Zap, Ambion, Austin, TX) to inactivate RNase enzymes.

Our experience in sequencing and assembling over six hundred transcriptomes also allows us to make conclusions and recommendations that promote implementation of NGS for the study of transcriptomes. Optimizing RNA integrity (RIN) and purity (OD 260/230) will improve size of assembled transcriptomes. However, we show that NGS is robust to variation in RNA quality, and therefore researchers and genome facilities should consider sequencing samples that show significant deviation from optimal levels of RIN scores, OD 260/230 ratios and certain other metrics of RNA quality. Implementing this recommendation could save researchers and sequencing facilities considerable time and money presently allocated for optimizing samples. Also, shipping dehydrated RNA to genome centers, instead of frozen extract, can further increase sequencing success.

### Conclusions

NGS is a powerful method for studying plant transcriptomes, and it is now possible to sequence virtually all expressed genes in a given tissue. Our study developed and evaluated many protocols that can facilitate the isolation of total RNA from most green plants. We have also identified several factors that affect the sequencing and assembly of transcriptomes. As a whole, these results provide a resource to increase success and efficiency of NGS to tackle the next generation of questions in plant biology.

## Supporting Information

Figure S1 **A comparison of the frequency of scaffolds according to variation in % GC content between samples sequenced on HiSeq versus GA II platforms.** Distributions are broadly overlapping. The long right-tail from the HiSeq samples is caused by a disproportionate number of algae samples sequenced on that platform. These algae exhibited especially rich GC transcripts, which is consistent with the results of published whole genome sequences of green algae [40], [41].
(PDF)

Table S1 **Raw data used to analyze RNA quality, sequencing depth and number of scaffolds.** For each sample we provide the species and family name, the tissue collected (Y-young, expanding tissue; M-mature, fully expanded but non-senescing tissue), the contributor of the sample, and the RNA isolation method (numbers correspond to Supplementary Protocols). Metrics of RNA quality and yield include the mass of total RNA isolated, RNA concentration, the ratio of the large RNA ribosomal subunit to the small RNA ribosomal subunit (r28S/18S), the RNA Integrity Number (RIN), OD 260/280, and OD 260/230. Sequencing data includes the sequencing platform, read length (averaged between the paired-ends), mass of total RNA sequenced, number of reads, number of bases, number of bases that surpassed the Q20 threshold and the number of scaffolds (c) greater than 100 bp (c100), greater than 200 bp (c200), and so on and so forth. We also include the number of scaffolds that fall within bins (b) of different sizes, ranging from b100 (i.e., the number of scaffolds 100–199 bp long) to b1000 (number of scaffolds greater 1000).
(XLS)

Table S2 **The success/failure of RNA isolations using Qiagen’s RNeasy Plant Minikit (see Appendix S1, Protocol 1) and alternative hybrid protocol (see Appendix S1, Protocol 2).** Successful isolations were those that met the stringent criteria of two isolations of the same tissue resulting in a concentration of >150 ng/µL, a total mass of 20 µg RNA, OD 260/280>1.9, OD 260/230>1.5, r26S/18S >1 and RIN >8. A ‘1’ denotes that the sample met these criteria and ‘0’ indicates that the sample did not meet these criteria; ‘−’ indicates the method was not attempted for the sample. In most cases, if a sample failed with the RNeasy kit (Appendix S1, Protocol 1) we attempted the alternative Qiagen recommended protocol (Appendix S1, Protocol 2). Family names are according to APG III (2009). Except when noted, we used freshly expanding leaves for all isolations.
(PDF)

Table S3 **Correlations in the number of scaffolds with different minimum threshold size cutoffs used during assemblies.** For example, the column labeled 500 bp contains all scaffolds that are 500 bp or larger. Pearson product moment correlation coefficients (*r*) are shown in the upper triangular matrix, and P-values are shown in the lower triangular matrix.
(PDF)

Table S4 **Least-squares means of descriptors of RNA quality among different plant tissues types.** The least-squares mean ±1 SE and sample size are provided for each tissue type.
(PDF)

Table S5 **P-values for a posteriori pairwise contrasts of RNA quality among tissue types.** P-values are adjusted for multiple comparisons within each variable using the Tukey-Kramer correction method. P-values <0.05 are bolded.
(PDF)

Table S6 **Least-squares means of descriptors of sequencing success among plant tissue types.** The least-squares mean ±1 SE and sample size are provided for each tissue type.
(PDF)

Table S7 **P-values for a posteriori pairwise contrasts of various sequencing metrics among different tissue types.** P-values are adjusted for multiple comparisons within each variable using the Tukey-Kramer correction method. P-values <0.05 are bolded.
(PDF)

Table S8 **The statistical fit of all possible combinations of explanatory factors in the data set that included OD ratios.** Fit of models was statistically compared using maximum likelihood statistics according to the Akaike information criterion (AIC). Models are arranged in the order of best-fitting models (lowest AIC) to poorer fitting models (higher AIC). Inclusion or absence of explanatory variables from models is shown by 1 and 0, respectively.
(PDF)

Table S9 **The statistical fit of all possible linear combinations of factors in the large data set that excluded OD ratios.** The fit of models was statistically compared using maximum likelihood statistics according to the Akaike information criterion (AIC). Models are arranged in the order of best-fitting models (lowest AIC) to poorer fitting models (higher AIC). Inclusion or absence of explanatory variables from models is shown by 1 and 0, respectively.
(PDF)

Appendix S1 **Eighteen protocols used to isolate total RNA from plant tissue included in this study.**
(PDF)
