# Supplementary material for: Evaluating Methods for Isolating Total RNA and Predicting the Success of Sequencing Phylogenetically Diverse Plant Transcriptomes
Source: PLoS One. 2012 Nov 21;7(11):e50226. doi: 10.1371/journal.pone.0050226 (PMC3504007; doi:10.1371/journal.pone.0050226)
Supplement: Figure S1 — A comparison of the frequency of scaffolds according to variation in % GC content between samples sequenced on HiSeq versus GA II platforms. Distributions are broadly overlapping. The long right-tail from the HiSeq samples is caused by a disproportionate number of algae samples sequenced on that platform. These algae exhibited especially rich GC transcripts, which is consistent with the results of published whole genome sequences of green algae [40], [41]. (PDF) [file pone.0050226.s001.pdf]

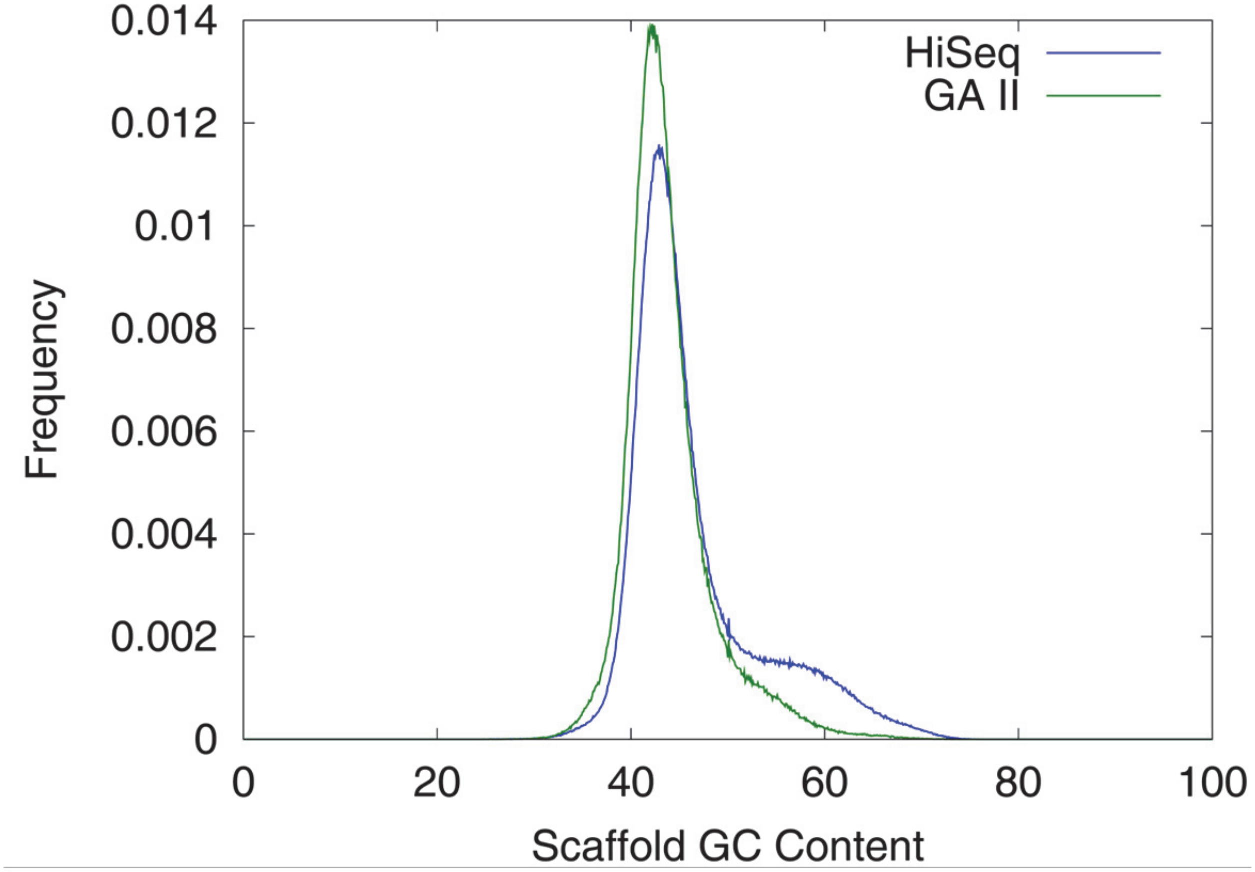

Figure S1. A comparison of the frequency of scaffolds according to variation in % GC content between samples sequenced on HiSeq versus GA II platforms. Distributions are broadly overlapping. The long right-tail from the HiSeq samples is caused by a disproportionate number of algae samples sequenced on that platform. These algae exhibited especially rich GC transcripts, which is consistent with the results of published whole genome sequences of green algae[40,41].
