# Supplementary material for: Evaluating Methods for Isolating Total RNA and Predicting the Success of Sequencing Phylogenetically Diverse Plant Transcriptomes
Source: PLoS One. 2012 Nov 21;7(11):e50226. doi: 10.1371/journal.pone.0050226 (PMC3504007; doi:10.1371/journal.pone.0050226)
Supplement: Table S2 — The success/failure of RNA isolations using Qiagen’s RNeasy Plant Minikit (see Appendix S1, Protocol 1) and alternative hybrid protocol (see Appendix S1, Protocol 2). Successful isolations were those that met the stringent criteria of two isolations of the same tissue resulting in a concentration of >150 ng/µL, a total mass of 20 µg RNA, OD 260/280>1.9, OD 260/230>1.5, r26S/18S >1 and RIN >8. A ‘1’ denotes that the sample met these criteria and ‘0’ indicates that the sample did not meet these criteria; ‘−’ indicates the method was not attempted for the sample. In most cases, if a sample failed with the RNeasy kit (Appendix S1, Protocol 1) we attempted the alternative Qiagen recommended protocol (Appendix S1, Protocol 2). Family names are according to APG III (2009). Except when noted, we used freshly expanding leaves for all isolations. (PDF) [file pone.0050226.s003.pdf]

Table S2 The success/failure of RNA isolations using Qiagen's RNeasy Plant Minikit (see Appendix S1, Protocol 1) and alternative hybrid protocol (see Appendix S1, Protocol 2). Successful isolations were those that met the stringent criteria of two isolations of the same tissue resulting in a concentration of > 150 ng/µL, a total mass of 20 µg RNA, OD 260/280 > 1.9, OD 260/230 > 1.5, r26S/18S > 1 and RIN > 8. A '1' denotes that the sample met these criteria and '0' indicates that the sample did not meet these criteria; '-' indicates the method was not attempted for the sample. In most cases, if a sample failed with the RNeasy kit (Appendix S1, Protocol 1) we attempted the alternative Qiagen recommended protocol (Appendix S1, Protocol 2). Family names are according to APG III (2009). Except when noted, we used freshly expanding leaves for all isolations.

**Family**	**Species**	**RNeasy**	**Alternative**
Actinidiaceae	*Actinidia chinensis*	1	-
Adoxaceae	*Viburnum* sp.	0	1
Asparagaceae	*Yucca brevifolia* ssp. *brevifolia*	1	-
Asparagaceae	*Yucca brevifolia* ssp. *jaegeriana*	1	-
Amaryllidaceae	*Phycella cyrtanthoides*	1	-
Amaryllidaceae	*Rhodophiala pratensis*	0	-
Amaryllidaceae	*Zephyranthes treatiae*	1	-
Symplocaceae	*Symplocus* sp.	1	-
Araliaceae	*Polyscias fruticos*	0	0
Arecaceae	*Serenoa repens*	0	0
Asteliaceae	*Astelia* (HYBRID)[1]	0	-
Bignoniaceae	*Mansoa alliacea*	1	-
Bignoniaceae	*Tabebuia umbellate*	1	-
Bromeliaceae	*Brocchinia reducta*	1	-
Cactaceae	*Pereskia aculeate*	0	0
Calycanthaceae	*Idiosporum australiense*	1	-
Cannabaceae	*Celtis occidentalis*	1	-
Cannaceae	*Canna* sp.	1	-
Caprifoliaceae	*Lonicera japonica*	1	-
Caryophyllaceae	*Schiedea membranacea*	1	-
Caryophyllaceae	*Schiedea salicaria*	0	0
Celastraceae	*Crossopetalum rhacoma*	0	0
Celastraceae	*Hippocratia parviflora*	0	0
Cephalotaceae	*Cephalotus follicularis*	0	0
Cephalotaxaceae	*Amentotaxus formosana*	0	0
Chrysobalanaceae	*Chrysobalanus icaco*	0	1
Cistaceae	*Cistus influtus*	0	0
Cornaceae	*Cornus floridana*	0	0
Cyrillaceae	*Cyrilla racemiflora*	1	-
Droseraceae	*Drosera capensis*	0	0
Elaeagnaceae	*Elaeagnus pungens*	0	-
Ericaceae	*Cavendishia cuatrecasasii*	0	1

Erythroyxlaceae	*Erythroxylon coca*	1	-
Fagaceae	*Quercus shumardii*	1	-
Fouquieriaceae	*Fouquieria macdougalii*	0	0
Gelsemiaceae	*Gelsemium semperivens*	1	-
Geraniaceae	*Geranium carolinianum*	1	-
Geraniaceae	*Geranium maculatum*[2]	0	1
Heliconiaceae	*Heliconia* sp.	1	-
Hydrangeaceae	*Hydrangea quercifolia*	1	-
Juglandaceae	*Carya glabra*	0	-
Lamiaceae	*Oxera neriifolia*	0	0
Lauraceae	*Sassafras albidum*	1	-
Lecythidaceae	*Barringtonia racemosa*	0	0
Malvaceae	*Hibiscus laevis*	0	0
Melanthiaceae	*Helonias bullata*	1	-
Melanthiaceae	*Xerophyllum asphodeloides*	1	-
Melastomataceae	*Medinilla magnifica*	0	1
Primulaceace	*Ardisia humilis*	0	0
Nyssaceae	*Nyssa ogeche*	0	1
Ochnaceae	*Ochna serrulata*	0	1
Olacaceae	*Ximenia Americana*	0	1
Oleaceae	*Chionanthus retusus*	1	-
Pandanaceae	*Freycinetia multiflora*	0	-
Papaveraceae	*Sanguinaria canadensis*	1	-
Petrosaviaceae	*Japonolirion osense*	1	-
Phytolaccaceae	*Petiveria allicea*	1	-
Picramniaceae	*Picramnia perfaundra*	1	-
Pittosporaceae	*Pittosporum sahnianum*	1	-
Plantaginaceae	*Plantago virginica*	0	-
Plumbaginaceae	*Plumbago auriculata*	0	0
Podocarpaceae	*Acmopyle pancheri*	0	0
Podocarpaceae	*Falcatifolium taxoides*	0	0
Podocarpaceae	*Sundacarpus amarus*	0	-
Polygonaceae	*Polygonella americana*	0	0
Rosaceae	*Rosa palustris*	0	0
Sapindaceae	*Aesculus pavia*	0	1
Sapindaceae	*Acer negundo*	1	-
Sapotaceae	*Sideroxylon reclinatum*	0	1
Sapotaceae	*Synsepalum dulcificum*	0	1
Sarraceniaceae	*Heliamphora minor*	0	0
Saururaceae	*Anemopsis californica*	0	0
Saxifragaceae	*Oresitrophe rupifraga*	0	1
Schisandraceae	*Illicium verum*	0	0
Solanaceae	*Brugmansia sanguinea*	1	-

Staphyleaceae	*Staphylea pinnata*	1	-
Stemonaceae	*Croomia* sp.	1	-
Styracaceae	*Sinojackia xylocarpa*	1	-
Thymelaeaceae	*Edgeworthia papyrifera*	1	-
Violaceae	*Viola tricolor*	0	0
Zingiberaceae	*Curcuma Olena*	1	-

[1] Meristematic bud tissue
[2] Mixture of leaves and flowers
