# Supplementary material for: Evaluating Methods for Isolating Total RNA and Predicting the Success of Sequencing Phylogenetically Diverse Plant Transcriptomes
Source: PLoS One. 2012 Nov 21;7(11):e50226. doi: 10.1371/journal.pone.0050226 (PMC3504007; doi:10.1371/journal.pone.0050226)
Supplement: Table S3 — Correlations in the number of scaffolds with different minimum threshold size cutoffs used during assemblies. For example, the column labeled 500 bp contains all scaffolds that are 500 bp or larger. Pearson product moment correlation coefficients (r) are shown in the upper triangular matrix, and P-values are shown in the lower triangular matrix. (PDF) [file pone.0050226.s004.pdf]

Table S3 Correlations in the number of scaffolds with different minimum threshold size cutoffs used during assemblies. For example, the column labeled 500 bp contains all scaffolds that are 500 bp or larger. Pearson product moment correlation coefficients (*r*) are shown in the upper triangular matrix, and P-values are shown in the lower triangular matrix.

	1000 bp	900 bp	800 bp	700 bp	600 bp	500 bp
1000 bp	1	0.99686	0.98475	0.95667	0.89861	0.78364
900 bp	<.0001	1	0.99531	0.97621	0.92897	0.82632
800 bp	<.0001	<.0001	1	0.99241	0.95957	0.87417
700 bp	<.0001	<.0001	<.0001	1	0.98666	0.92595
600 bp	<.0001	<.0001	<.0001	<.0001	1	0.97468
500 bp	<.0001	<.0001	<.0001	<.0001	<.0001	1
