# Supplementary material for: Evaluating Methods for Isolating Total RNA and Predicting the Success of Sequencing Phylogenetically Diverse Plant Transcriptomes
Source: PLoS One. 2012 Nov 21;7(11):e50226. doi: 10.1371/journal.pone.0050226 (PMC3504007; doi:10.1371/journal.pone.0050226)
Supplement: Table S4 — Least-squares means of descriptors of RNA quality among different plant tissues types. The least-squares mean ±1 SE and sample size are provided for each tissue type. (PDF) [file pone.0050226.s005.pdf]

Table S4 Least-squares means of descriptors of RNA quality among different plant tissues types. The least-squares mean ± 1 SE and sample size are provided for each tissue type.

	$\log_e$(RNA conc.)		26S:18S		RIN		OD 260/280		OD 260/230	
**Tissue type**	**Mean**	**N**	**Mean**	**N**	**Mean**	**N**	**Mean**	**N**	**Mean**	**N**
Belowground	3.67 ± 0.08	12	1.43 ± 0.18	12	7.49 ± 7.49	12	1.57 + 0.33	1	0.91 + 0.64	1
Shoots/Stems	3.58 ± 0.09	7	1.50 ± 0.24	7	6.24 ± 6.24	7	1.98 + 0.33	1	1.93 + 0.64	1
Buds (lvs[a]/flws[b])	3.65 ± 0.22	15	1.26 ± 0.16	15	6.75 ± 6.75	15	1.73 + 0.14	6	1.38 + 0.26	6
Leaf	3.53 ± 0.05	492	1.15 ± 0.03	489	6.21 ± 6.21	480	1.97 + 0.02	378	1.69 + 0.04	258
Flower	3.96 ± 0.34	4	1.00 ± 0.31	4	4.68 ± 4.68	4	2.07 + 0.24	2	1.40 + 0.46	2
Fruit	2.73 ± 0.34	10	1.55 ± 0.20	10	7.41 ± 7.41	10	-	0	-	0
Mixed tissue	3.67 ± 0.07	276	1.07 ± 0.04	276	5.99 ± 5.99	273	1.87 + 0.03	121	1.55 + 0.06	121
Algal cells	3.30 ± 0.06	274	1.47 ± 0.04	168	6.75 ± 6.75	268	1.03 + 0.33	1	0.94 + 0.64	1

[a]leaves. [b]flowers.
