# Supplementary material for: Evaluating Methods for Isolating Total RNA and Predicting the Success of Sequencing Phylogenetically Diverse Plant Transcriptomes
Source: PLoS One. 2012 Nov 21;7(11):e50226. doi: 10.1371/journal.pone.0050226 (PMC3504007; doi:10.1371/journal.pone.0050226)
Supplement: Table S5 — P-values for a posteriori pairwise contrasts of RNA quality among tissue types. P-values are adjusted for multiple comparisons within each variable using the Tukey-Kramer correction method. P-values <0.05 are bolded. (PDF) [file pone.0050226.s006.pdf]

Table S5 P-values for a posteriori pairwise contrasts of RNA quality among tissue types. P-values are adjusted for multiple comparisons within each variable using the Tukey-Kramer correction method. P-values < 0.05 are bolded.

		P-values				
**Tissue 1**	**Tissue 2**	**RNA mass**	**r26S:18S**	**RIN**	**OD 260/280**	**OD 260/230**
Belowground	Buds (lvs[a]/flws[b])	0.9344	0.9974	0.9702	0.9995	0.9942
Belowground	Algal cells	**0.0015**	1	0.8795	0.9137	1
Belowground	Flower	0.4724	0.9368	0.1542	0.8845	0.9961
Belowground	Fruit	**0.0234**	0.9998	1	-	-
Belowground	Leaf	0.1685	0.815	0.2662	0.8989	0.8912
Belowground	Mixed tissue	0.9602	0.5124	0.1195	0.9723	0.9561
Belowground	Shoot/Stem	0.4701	1	0.8558	0.9769	0.9218
Buds (lvs/flws)	Algal cells	0.1538	0.9157	1	0.4615	0.996
Buds (lvs/flws)	Flower	0.4849	0.9957	0.5044	0.8666	1
Buds (lvs/flws)	Fruit	**0.0386**	0.9475	0.9887	-	-
Buds (lvs/flws)	Leaf	0.6048	0.9982	0.9561	0.5763	0.9012
Buds (lvs/flws)	Mixed tissue	0.9149	0.9388	0.7985	0.9415	0.995
Buds (lvs/flws)	Shoot/Stem	0.7720	0.9906	0.999	0.9921	0.9851
Algal cells	Flower	0.1529	0.8133	0.3536	0.1452	0.9973
Algal cells	Fruit	0.1314	0.9999	0.9567	-	-
Algal cells	Leaf	**0.0038**	**<0.0001**	**0.0039**	0.0766	0.9083
Algal cells	Mixed tissue	**<0.0001**	**<0.0001**	**<0.0001**	0.1565	0.9652
Algal cells	Shoot/Stem	**0.0233**	1	0.9969	0.4066	0.9317
Flower	Fruit	**0.0326**	0.8107	0.2091	-	-
Flower	Leaf	0.3029	0.9997	0.7327	0.9995	0.9957
Flower	Mixed tissue	0.4781	1	0.8575	0.9813	0.9999
Flower	Shoot/Stem	0.3578	0.9055	0.8846	1	0.994
Fruit	Leaf	**0.0453**	0.4903	0.4718	-	-
Fruit	Mixed tissue	**0.0225**	0.2346	0.2674	-	-
Fruit	Shoot/Stem	**0.0369**	1	0.9112	-	-
Leaf	Mixed tissue	0.0874	0.558	0.8139	0.0955	0.4422
Leaf	Shoot/Stem	0.6345	0.8298	1	1	0.9998
Mixed tissue	Shoot/Stem	0.4037	0.6051	1	0.9999	0.9971

[a]leaves. [b]flowers.
