# Supplementary material for: Evaluating Methods for Isolating Total RNA and Predicting the Success of Sequencing Phylogenetically Diverse Plant Transcriptomes
Source: PLoS One. 2012 Nov 21;7(11):e50226. doi: 10.1371/journal.pone.0050226 (PMC3504007; doi:10.1371/journal.pone.0050226)
Supplement: Table S6 — Least-squares means of descriptors of sequencing success among plant tissue types. The least-squares mean ±1 SE and sample size are provided for each tissue type. (PDF) [file pone.0050226.s007.pdf]

Table S6 Least-squares means of descriptors of sequencing success among plant tissue types. The least-squares mean ± 1 SE and sample size are provided for each tissue type.

	Bases		Q20		Reads		Scaffolds	
**Tissue type**	**Mean**	**N**	**Mean**	**N**	**Mean**	**N**	**Mean**	**N**
Belowground	2.12 E9 ± 1.25 E8	13	2.05 E9 ± 1.26 E8	13	1.30 E7 ± 0.73	13	5983 ± 687	14
Shoots/Stems	1.76 E9 ± 1.36 E8	11	1.62 E9 ± 1.37 E8	11	1.19 E7 ± 0.80	11	6356 ± 747	11
Buds (lvs[a]/flws[b])	2.15 E9 ± 1.50 E8	9	2.07 E9 ± 1.52 E8	9	1.43 E7 ± 0.88	9	6761 ± 826	10
Leaf	2.04 E9 ± 0.26 E8	304	1.96 E9 ± 0.26 E8	304	1.18 E7 ± 0.15	304	4826 ± 143	380
Flower	2.45 E9 ± 3.19 E8	2	2.37 E9 ± 3.22 E8	2	1.51 E7 ± 1.87	2	6437 ± 1751	2
Fruit	2.21 E9 ± 2.02 E8	5	2.13 E9 ± 2.03 E8	5	1.40 E7 ± 1.18	5	6816 ± 1108	5
Mixed tissue	2.02 E9 ± 0.36 E8	157	1.93 E9 ± 0.36 E8	157	1.17 E7 ± 0.21	157	5124 ± 198	206
Algal cells	1.67 E9 ± 0.50 E8	83	1.46 E9 ± 0.50 E8	83	0.95 E7 ± 0.29	83	4709 ± 272	248

[a]leaves. [b]flowers.
