# Supplementary material for: Evaluating Methods for Isolating Total RNA and Predicting the Success of Sequencing Phylogenetically Diverse Plant Transcriptomes
Source: PLoS One. 2012 Nov 21;7(11):e50226. doi: 10.1371/journal.pone.0050226 (PMC3504007; doi:10.1371/journal.pone.0050226)
Supplement: Table S7 — P-values for a posteriori pairwise contrasts of various sequencing metrics among different tissue types. P-values are adjusted for multiple comparisons within each variable using the Tukey-Kramer correction method. P-values <0.05 are bolded. (PDF) [file pone.0050226.s008.pdf]

Table S7 P-values for a posteriori pairwise contrasts of various sequencing metrics among different tissue types. P-values are adjusted for multiple comparisons within each variable using the Tukey-Kramer correction method. P-values < 0.05 are bolded.

		P-values			
**Tissue 1**	**Tissue 2**	**Bases**	**Q20**	**Reads**	**Scaffolds**
Belowground	Buds (lvs[a]/flws[b])	1	1	0.9529	0.9963
Belowground	Algal cells	**0.0189**	**0.0004**	**0.0002**	0.6716
Belowground	Flower	0.9679	0.9845	0.9694	1
Belowground	Fruit	0.9999	1	0.9956	0.9983
Belowground	Leaf	0.9987	0.9972	0.7556	0.7201
Belowground	Mixed tissue	0.9958	0.9832	0.6628	0.9313
Belowground	Shoot/Stem	0.5426	0.2742	0.9683	1
Buds (lvs/flws)	Algal cells	**0.0462**	**0.0039**	**<.0001**	0.2634
Buds (lvs/flws)	Flower	0.9846	0.9909	0.9999	1
Buds (lvs/flws)	Fruit	1	1	1	1
Buds (lvs/flws)	Leaf	0.9959	0.997	0.1059	0.2904
Buds (lvs/flws)	Mixed tissue	0.9906	0.9859	0.0792	0.5321
Buds (lvs/flws)	Shoot/Stem	0.5435	0.3394	0.4655	1
Algal cells	Flower	0.1952	0.1019	0.0616	0.9779
Algal cells	Fruit	0.1469	**0.0308**	**0.0046**	0.588
Algal cells	Leaf	**<.0001**	**<.0001**	**<.0001**	0.9999
Algal cells	Mixed tissue	**<.0001**	**<.0001**	**<.0001**	0.9217
Algal cells	Shoot/Stem	0.9975	0.9671	0.0852	0.4345
Flower	Fruit	0.9971	0.9987	0.9998	1
Flower	Leaf	0.8753	0.9141	0.6604	0.9845
Flower	Mixed tissue	0.8522	0.8772	0.6157	0.9956
Flower	Shoot/Stem	0.45	0.383	0.7658	1
Fruit	Leaf	0.9901	0.9909	0.5738	0.6328
Fruit	Mixed tissue	0.9829	0.9758	0.5064	0.8054
Fruit	Shoot/Stem	0.5912	0.4074	0.7993	1
Leaf	Mixed tissue	0.9999	0.9959	0.9996	0.9252
Leaf	Shoot/Stem	0.4899	0.2041	1	0.4744
Mixed tissue	Shoot/Stem	0.5999	0.3455	1	0.7534

[a]leaves. [b]flowers.
