# Supplementary material for: Evaluating Methods for Isolating Total RNA and Predicting the Success of Sequencing Phylogenetically Diverse Plant Transcriptomes
Source: PLoS One. 2012 Nov 21;7(11):e50226. doi: 10.1371/journal.pone.0050226 (PMC3504007; doi:10.1371/journal.pone.0050226)
Supplement: Table S8 — The statistical fit of all possible combinations of explanatory factors in the data set that included OD ratios. Fit of models was statistically compared using maximum likelihood statistics according to the Akaike information criterion (AIC). Models are arranged in the order of best-fitting models (lowest AIC) to poorer fitting models (higher AIC). Inclusion or absence of explanatory variables from models is shown by 1 and 0, respectively. (PDF) [file pone.0050226.s009.pdf]

Table S8 The statistical fit of all possible combinations of explanatory factors in the data set that included OD ratios. Fit of models was statistically compared using maximum likelihood statistics according to the Akaike information criterion (AIC). Models are arranged in the order of best-fitting models (lowest AIC) to poorer fitting models (higher AIC). Inclusion or absence of explanatory variables from models is shown by 1 and 0, respectively.

Tissue	RNA conc.	r26S:18S	RIN	OD 260/280	OD 260/230	Platform	µg RNA sequenced	Bases sequenced	AIC
1	0	1	1	1	1	1	1	0	3187
1	1	1	1	1	1	1	1	0	3187.8
1	0	0	1	1	1	1	1	0	3201.8
1	1	0	1	1	1	1	1	0	3202.6
1	0	1	1	1	1	1	0	0	3202.6
1	1	1	1	0	1	1	1	0	3204.3
1	0	1	1	0	1	1	1	0	3204.8
1	1	1	1	1	1	1	0	0	3206.1
1	0	1	0	1	1	1	1	0	3208.1
1	1	1	0	1	1	1	1	0	3209.7
1	0	1	1	1	1	1	1	1	3214.5
1	0	1	1	1	0	1	1	0	3214.6
1	1	1	1	1	0	1	1	0	3215.7
1	1	1	1	1	1	1	1	1	3215.7
0	0	1	1	1	1	1	1	0	3218.1
1	1	0	1	0	1	1	1	0	3219
1	0	0	1	1	1	1	0	0	3219.1
1	0	1	1	0	1	1	0	0	3219.2
0	1	1	1	1	1	1	1	0	3219.2
1	0	0	1	0	1	1	1	0	3219.6
1	0	1	0	1	1	1	0	0	3220.9
1	1	1	1	0	1	1	0	0	3222.5
1	1	0	1	1	1	1	0	0	3222.7
1	1	1	0	1	1	1	0	0	3224.4
1	0	1	1	1	0	1	0	0	3224.9
1	0	1	0	0	1	1	1	0	3226
1	1	1	0	0	1	1	1	0	3226.4
1	1	1	1	1	0	1	0	0	3228.1
1	0	1	1	0	0	1	1	0	3228.8
1	0	0	1	1	1	1	1	1	3229.2
1	1	1	1	0	0	1	1	0	3230.2
1	1	0	1	1	1	1	1	1	3230.4
1	0	0	1	1	0	1	1	0	3230.7
1	0	1	1	1	1	1	0	1	3230.8
1	1	0	1	1	0	1	1	0	3231.7
1	0	1	0	1	0	1	1	0	3232.2
1	1	1	1	0	1	1	1	1	3232.3

1	0	1	1	0	1	1	1	1	3232.6
0	0	0	1	1	1	1	1	0	3233
1	1	1	0	1	0	1	1	0	3233.7
0	1	0	1	1	1	1	1	0	3234.1
1	1	1	1	1	1	1	0	1	3234.2
0	0	1	1	1	1	1	0	0	3234.4
1	0	0	1	0	1	1	0	0	3235.4
1	0	1	0	1	1	1	1	1	3235.4
1	0	0	0	1	1	1	1	0	3235.5
0	1	1	1	0	1	1	1	0	3235.7
0	0	1	1	0	1	1	1	0	3235.8
1	1	1	0	1	1	1	1	1	3237.3
1	1	0	0	1	1	1	1	0	3237.6
1	0	1	0	0	1	1	0	0	3237.8
0	1	1	1	1	1	1	0	0	3238
1	1	0	1	0	1	1	0	0	3238.9
1	0	1	1	0	0	1	0	0	3239
0	0	1	0	1	1	1	1	0	3239.3
1	1	1	0	0	1	1	0	0	3241
0	1	1	0	1	1	1	1	0	3241.1
1	0	1	0	1	0	1	0	0	3241.5
1	0	0	1	1	0	1	0	0	3242
1	1	1	1	0	0	1	0	0	3242.3
1	0	1	1	1	0	1	1	1	3242.7
1	1	1	1	1	0	1	1	1	3243.7
1	1	1	0	1	0	1	0	0	3244.7
1	0	0	1	0	0	1	1	0	3245
1	1	0	1	1	0	1	0	0	3245.4
0	0	1	1	1	1	1	1	1	3245.6
1	0	1	0	0	0	1	1	0	3246.3
0	0	1	1	1	0	1	1	0	3246.3
1	1	0	1	0	0	1	1	0	3246.6
1	1	0	1	0	1	1	1	1	3247
0	1	1	1	1	1	1	1	1	3247.1
1	0	0	1	0	1	1	1	1	3247.3
1	0	1	1	0	1	1	0	1	3247.3
1	0	0	1	1	1	1	0	1	3247.3
0	1	1	1	1	0	1	1	0	3247.7
1	1	1	0	0	0	1	1	0	3248
1	0	1	0	1	1	1	0	1	3249.1
1	1	1	1	0	1	1	0	1	3250.5
0	1	0	1	0	1	1	1	0	3250.6
0	0	0	1	0	1	1	1	0	3250.7

1	1	0	1	1	1	1	0	1	3250.7
0	0	1	1	0	1	1	0	0	3250.9
1	0	0	0	1	1	1	0	0	3251
0	0	0	1	1	1	1	0	0	3251.3
1	0	1	1	1	1	0	1	0	3251.9
1	1	1	0	1	1	1	0	1	3252.5
0	0	1	0	1	1	1	0	0	3252.7
1	0	1	1	1	0	1	0	1	3252.9
1	0	0	0	0	1	1	1	0	3253.2
1	0	1	0	0	1	1	1	1	3253.6
1	1	0	0	1	1	1	0	0	3254.3
1	1	1	0	0	1	1	1	1	3254.3
0	1	1	1	0	1	1	0	0	3254.4
1	1	0	0	0	1	1	1	0	3254.4
1	1	1	1	1	1	0	1	0	3254.8
0	1	0	1	1	1	1	0	0	3254.8
1	0	1	0	0	0	1	0	0	3255.6
1	1	1	1	1	0	1	0	1	3255.9
0	1	1	0	1	1	1	0	0	3256.2
1	0	0	1	0	0	1	0	0	3256.3
0	0	1	1	1	0	1	0	0	3256.9
1	0	1	1	0	0	1	1	1	3256.9
0	0	1	0	0	1	1	1	0	3257.2
0	1	1	0	0	1	1	1	0	3257.9
1	1	1	1	0	0	1	1	1	3258.3
1	1	1	0	0	0	1	0	0	3258.8
1	0	0	1	1	0	1	1	1	3258.8
1	1	0	1	0	0	1	0	0	3259.7
1	1	0	1	1	0	1	1	1	3259.8
1	0	0	0	1	0	1	1	0	3260.1
1	0	1	0	1	0	1	1	1	3260.1
0	1	1	1	1	0	1	0	0	3260.3
0	0	1	1	0	0	1	1	0	3260.4
0	0	0	1	1	1	1	1	1	3260.4
1	1	1	0	1	0	1	1	1	3261.7
1	0	0	0	1	1	1	1	1	3261.7
0	1	0	1	1	1	1	1	1	3261.9
1	1	0	0	1	0	1	1	0	3262.1
0	1	1	1	0	0	1	1	0	3262.3
0	0	0	1	1	0	1	1	0	3262.6
0	0	1	1	1	1	1	0	1	3262.6
0	0	1	1	0	1	1	1	1	3263.6
1	0	0	1	0	1	1	0	1	3263.6

0	1	1	1	0	1	1	1	1	3263.7
0	0	1	0	1	0	1	1	0	3263.8
0	1	0	1	1	0	1	1	0	3264
1	1	0	0	1	1	1	1	1	3264.2
0	1	1	0	1	0	1	1	0	3265.7
1	0	1	0	0	1	1	0	1	3265.9
0	1	1	1	1	1	1	0	1	3266
0	0	1	0	1	1	1	1	1	3266.6
1	1	0	1	0	1	1	0	1	3266.9
1	0	1	1	0	0	1	0	1	3267.1
1	0	0	0	0	1	1	0	0	3267.4
0	0	0	0	1	1	1	1	0	3267.4
0	0	0	1	0	1	1	0	0	3267.5
1	0	0	1	1	1	0	1	0	3268.2
0	1	1	0	1	1	1	1	1	3268.7
1	1	1	0	0	1	1	0	1	3269.1
0	0	1	0	0	1	1	0	0	3269.4
1	0	1	0	1	0	1	0	1	3269.6
0	1	0	0	1	1	1	1	0	3269.8
1	0	0	1	1	0	1	0	1	3270.1
1	1	1	1	0	0	1	0	1	3270.2
1	0	1	1	0	1	0	1	0	3270.4
1	1	0	0	0	1	1	0	0	3270.9
0	0	1	1	0	0	1	0	0	3271
1	0	0	0	1	0	1	0	0	3271
0	1	0	1	0	1	1	0	0	3271.1
1	1	0	1	1	1	0	1	0	3271.2
1	0	1	0	1	1	0	1	0	3272.3
1	1	1	0	1	0	1	0	1	3272.7
1	1	1	1	0	1	0	1	0	3272.9
0	1	1	0	0	1	1	0	0	3272.9
1	0	1	1	1	1	0	0	0	3273
1	0	0	1	0	0	1	1	1	3273
1	1	0	1	1	0	1	0	1	3273.3
0	0	1	0	1	0	1	0	0	3273.4
1	0	0	0	0	0	1	1	0	3274.2
1	0	1	0	0	0	1	1	1	3274.3
0	0	1	1	1	0	1	1	1	3274.3
1	1	0	0	1	0	1	0	0	3274.4
1	1	1	1	1	1	0	0	0	3274.4
0	1	1	1	0	0	1	0	0	3274.5
0	0	0	1	1	0	1	0	0	3274.5
1	1	0	1	0	0	1	1	1	3274.6

1	1	1	0	1	1	0	1	0	3275.4
0	1	1	1	1	0	1	1	1	3275.7
1	1	1	0	0	0	1	1	1	3276
1	1	0	0	0	0	1	1	0	3276.5
0	1	1	0	1	0	1	0	0	3276.8
0	0	0	1	0	0	1	1	0	3276.9
0	0	1	0	0	0	1	1	0	3277.9
0	1	0	1	1	0	1	0	0	3278
0	0	0	1	0	1	1	1	1	3278.4
0	1	0	1	0	1	1	1	1	3278.5
1	0	0	0	1	1	1	0	1	3278.8
0	1	0	1	0	0	1	1	0	3278.9
0	0	1	1	0	1	1	0	1	3279
1	0	1	1	1	1	0	1	1	3279.4
0	0	0	1	1	1	1	0	1	3279.5
1	0	0	0	0	1	1	1	1	3279.8
0	1	1	0	0	0	1	1	0	3280
0	0	1	0	1	1	1	0	1	3280.8
1	1	0	0	0	1	1	1	1	3281.4
1	1	0	0	1	1	1	0	1	3281.9
0	1	1	1	0	1	1	0	1	3282.4
1	1	1	1	1	1	0	1	1	3282.4
0	1	0	1	1	1	1	0	1	3282.9
0	0	1	1	1	1	0	1	0	3283.2
1	0	1	0	0	0	1	0	1	3283.7
0	0	0	0	1	1	1	0	0	3283.9
1	0	0	1	0	0	1	0	1	3284.3
0	1	1	0	1	1	1	0	1	3284.3
0	0	1	0	0	1	1	1	1	3284.8
0	0	1	1	1	0	1	0	1	3284.9
0	0	0	0	0	1	1	1	0	3285
1	0	0	0	0	0	1	0	0	3285.2
0	1	1	0	0	1	1	1	1	3285.8
0	1	1	1	1	1	0	1	0	3286.1
0	1	0	0	0	1	1	1	0	3286.6
1	0	0	1	0	1	0	1	0	3286.6
1	1	1	0	0	0	1	0	1	3286.8
0	1	0	0	1	1	1	0	0	3287
1	0	0	0	1	0	1	1	1	3287.4
0	0	1	0	0	0	1	0	0	3287.5
1	1	0	1	0	0	1	0	1	3287.7
1	0	1	1	1	0	0	1	0	3287.9
0	1	1	1	1	0	1	0	1	3288.2

0	0	1	1	0	0	1	1	1	3288.5
1	1	0	0	0	0	1	0	0	3288.7
0	0	0	1	0	0	1	0	0	3288.8
1	1	0	1	0	1	0	1	0	3289.1
1	1	0	0	1	0	1	1	1	3289.7
1	0	1	1	0	1	0	0	0	3289.8
1	0	1	0	1	1	0	0	0	3290.1
0	1	1	1	0	0	1	1	1	3290.4
0	0	0	1	1	0	1	1	1	3290.6
1	1	1	1	1	0	0	1	0	3290.8
0	1	1	0	0	0	1	0	0	3291
1	0	1	0	0	1	0	1	0	3291
1	1	1	0	1	1	0	0	0	3291.6
0	0	1	0	1	0	1	1	1	3291.8
0	1	0	1	1	0	1	1	1	3292.1
1	1	1	1	0	1	0	0	0	3292.3
0	1	0	1	0	0	1	0	0	3292.4
0	0	0	0	1	0	1	1	0	3292.5
1	0	0	1	1	1	0	0	0	3292.8
1	1	0	1	1	1	0	0	0	3293.4
0	0	0	0	1	1	1	1	1	3293.5
1	1	1	0	0	1	0	1	0	3293.7
0	1	1	0	1	0	1	1	1	3293.7
0	1	0	0	1	0	1	1	0	3294.9
1	0	0	0	0	1	1	0	1	3295.3
0	0	0	1	0	1	1	0	1	3295.6
1	0	0	1	1	1	0	1	1	3295.6
0	1	0	0	1	1	1	1	1	3296.2
0	0	1	0	0	1	1	0	1	3297.6
1	0	1	1	0	1	0	1	1	3298.1
1	1	0	1	1	1	0	1	1	3298.6
1	1	0	0	0	1	1	0	1	3298.7
1	0	0	0	1	0	1	0	1	3299
0	0	1	1	0	0	1	0	1	3299.1
0	1	0	1	0	1	1	0	1	3299.1
0	0	0	1	1	1	0	1	0	3299.6
1	0	1	0	1	1	0	1	1	3299.7
0	0	0	0	0	1	1	0	0	3300.1
1	0	1	1	1	0	0	0	0	3300.5
1	0	1	1	1	1	0	0	1	3300.5
1	1	1	1	0	1	0	1	1	3300.6
0	1	1	0	0	1	1	0	1	3301
1	0	0	0	0	0	1	1	1	3301.6

0	0	1	0	1	0	1	0	1	3301.6
0	0	1	1	0	1	0	1	0	3301.7
1	1	1	1	1	1	0	0	1	3302.1
1	1	0	0	1	0	1	0	1	3302.3
0	1	1	1	0	0	1	0	1	3302.4
0	1	0	1	1	1	0	1	0	3302.5
0	0	0	1	1	0	1	0	1	3302.5
1	1	1	0	1	1	0	1	1	3302.7
1	0	1	1	0	0	0	1	0	3302.8
1	1	1	1	1	0	0	0	0	3302.9
0	1	0	0	0	1	1	0	0	3303.6
0	0	1	0	1	1	0	1	0	3303.7
0	0	0	0	1	0	1	0	0	3304.1
0	1	1	1	0	1	0	1	0	3304.1
1	1	0	0	0	0	1	1	1	3304.1
1	0	1	0	1	0	0	1	0	3304.2
0	0	1	1	1	1	0	0	0	3304.4
1	0	0	0	1	1	0	1	0	3304.5
0	1	1	0	1	0	1	0	1	3304.8
0	0	0	1	0	0	1	1	1	3304.9
0	1	1	1	1	1	0	0	0	3305.8
1	1	1	1	0	0	0	1	0	3305.9
0	0	1	0	0	0	1	1	1	3305.9
0	1	0	1	1	0	1	0	1	3305.9
0	0	0	0	0	0	1	1	0	3306.7
0	1	1	0	1	1	0	1	0	3306.7
1	0	0	1	1	0	0	1	0	3306.8
0	1	0	1	0	0	1	1	1	3306.9
1	1	1	0	1	0	0	1	0	3307.1
1	0	1	0	0	1	0	0	0	3307.2
1	1	0	0	1	1	0	1	0	3307.4
0	1	0	0	1	0	1	0	0	3307.5
0	1	1	0	0	0	1	1	1	3308
1	0	0	1	0	1	0	0	0	3309.3
0	1	0	0	0	0	1	1	0	3309.4
1	1	1	0	0	1	0	0	0	3309.7
1	1	0	1	1	0	0	1	0	3309.8
0	0	1	1	1	1	0	1	1	3310.7
1	1	0	1	0	1	0	0	0	3311.2
0	0	0	0	0	1	1	1	1	3311.6
0	0	0	0	1	1	1	0	1	3311.8
1	0	0	0	0	0	1	0	1	3313.2
0	1	0	0	0	1	1	1	1	3313.5

0	1	1	1	1	1	0	1	1	3313.7
1	0	0	1	0	1	0	1	1	3314.2
0	1	0	0	1	1	1	0	1	3314.6
1	0	1	1	0	0	0	0	0	3315.4
1	0	1	1	1	0	0	1	1	3315.6
0	0	1	0	0	0	1	0	1	3315.7
1	0	1	0	1	0	0	0	0	3315.8
1	1	0	0	0	0	1	0	1	3316.6
0	0	0	1	0	0	1	0	1	3316.8
1	1	0	1	0	1	0	1	1	3316.8
1	0	1	1	0	1	0	0	1	3317.3
1	1	1	1	0	0	0	0	0	3317.6
1	0	1	0	1	1	0	0	1	3317.8
0	0	0	1	0	1	0	1	0	3318
1	1	1	0	1	0	0	0	0	3318.2
0	0	0	0	0	0	1	0	0	3318.3
1	1	1	1	1	0	0	1	1	3318.4
1	0	1	0	0	1	0	1	1	3318.5
1	0	1	0	0	0	0	1	0	3318.8
0	1	1	0	0	0	1	0	1	3319
0	0	1	1	1	0	0	1	0	3319.3
1	1	1	0	1	1	0	0	1	3319.3
0	0	0	0	1	0	1	1	1	3319.9
1	1	1	1	0	1	0	0	1	3319.9
0	1	0	1	0	0	1	0	1	3320.3
0	1	0	1	0	1	0	1	0	3320.4
1	0	0	1	1	1	0	0	1	3320.4
1	1	0	1	1	1	0	0	1	3321.1
0	0	1	1	0	1	0	0	0	3321.2
1	1	1	0	0	1	0	1	1	3321.3
0	0	1	0	1	1	0	0	0	3321.5
0	1	0	0	0	0	1	0	0	3321.8
1	0	0	1	1	0	0	0	0	3321.8
1	1	1	0	0	0	0	1	0	3321.9
1	0	0	1	0	0	0	1	0	3322.2
0	1	1	1	1	0	0	1	0	3322.2
0	0	1	0	0	1	0	1	0	3322.3
0	1	0	0	1	0	1	1	1	3322.5
1	0	0	0	0	1	0	1	0	3322.8
0	1	1	0	1	1	0	0	0	3323
1	1	0	1	1	0	0	0	0	3323.6
0	1	1	1	0	1	0	0	0	3323.7
0	0	0	1	1	1	0	0	0	3324.4

0	1	0	1	1	1	0	0	0	3324.9
0	1	1	0	0	1	0	1	0	3325
1	1	0	1	0	0	0	1	0	3325.3
1	1	0	0	0	1	0	1	0	3325.8
1	1	0	0	1	1	0	0	0	3326.1
1	0	0	0	1	1	0	0	0	3326.8
0	0	0	1	1	1	0	1	1	3326.9
1	0	1	1	1	0	0	0	1	3327.7
0	0	0	0	0	1	1	0	1	3328.1
0	0	1	1	0	1	0	1	1	3329.4
0	1	0	1	1	1	0	1	1	3329.9
1	1	1	1	1	0	0	0	1	3330.3
1	0	1	0	0	0	0	0	0	3330.4
1	0	1	1	0	0	0	1	1	3330.5
1	0	0	0	1	1	0	1	1	3330.6
0	0	1	0	1	1	0	1	1	3331
0	1	0	0	0	1	1	0	1	3331.5
1	0	1	0	1	0	0	1	1	3331.9
0	1	1	1	0	1	0	1	1	3331.9
0	0	1	1	1	1	0	0	1	3331.9
0	0	1	1	1	0	0	0	0	3332
0	0	0	0	1	0	1	0	1	3332.1
1	1	1	0	0	0	0	0	0	3332.7
1	1	0	0	1	1	0	1	1	3333.4
1	1	1	1	0	0	0	1	1	3333.5
0	1	1	1	1	1	0	0	1	3333.5
0	1	1	0	1	1	0	1	1	3334
0	0	0	0	0	0	1	1	1	3334.1
0	0	1	1	0	0	0	1	0	3334.2
0	1	1	1	1	0	0	0	0	3334.4
1	0	0	1	1	0	0	1	1	3334.6
1	1	1	0	1	0	0	1	1	3334.8
1	0	1	0	0	1	0	0	1	3334.9
0	1	0	0	1	0	1	0	1	3335.4
0	0	1	0	1	0	0	1	0	3335.6
0	0	0	0	1	1	0	1	0	3336
1	0	0	1	0	1	0	0	1	3336.8
0	1	0	0	0	0	1	1	1	3336.9
1	0	0	1	0	0	0	0	0	3337.1
0	1	1	1	0	0	0	1	0	3337.3
1	1	0	1	1	0	0	1	1	3337.4
1	1	1	0	0	1	0	0	1	3337.5
1	0	0	0	1	0	0	1	0	3337.7

0	0	0	1	1	0	0	1	0	3338.4
1	1	0	1	0	0	0	0	0	3338.5
0	1	1	0	1	0	0	1	0	3338.6
0	0	1	0	0	1	0	0	0	3338.6
1	1	0	1	0	1	0	0	1	3338.8
0	1	0	0	1	1	0	1	0	3338.9
1	1	0	0	1	0	0	1	0	3340.4
0	0	0	1	0	1	0	0	0	3340.9
0	1	1	0	0	1	0	0	0	3341.1
0	1	0	1	1	0	0	1	0	3341.3
1	0	1	1	0	0	0	0	1	3342.5
0	1	0	1	0	1	0	0	0	3342.6
1	0	1	0	1	0	0	0	1	3343.2
1	0	0	0	0	1	0	0	0	3343.5
1	1	0	0	0	1	0	0	0	3344.3
1	1	1	1	0	0	0	0	1	3345
0	0	0	1	0	1	0	1	1	3345.6
1	1	1	0	1	0	0	0	1	3345.8
0	0	0	0	0	0	1	0	1	3346.4
1	0	1	0	0	0	0	1	1	3346.6
0	0	1	1	0	0	0	0	0	3346.9
0	0	1	1	1	0	0	1	1	3347
0	0	1	0	1	0	0	0	0	3347.3
0	1	0	1	0	1	0	1	1	3348.1
0	0	1	1	0	1	0	0	1	3348.6
1	0	0	1	1	0	0	0	1	3349
0	1	1	1	0	0	0	0	0	3349.1
0	0	1	0	1	1	0	0	1	3349.2
1	0	0	0	0	1	0	1	1	3349.5
1	1	1	0	0	0	0	1	1	3349.6
0	1	1	0	1	0	0	0	0	3349.7
0	1	0	0	0	0	1	0	1	3349.7
0	1	1	1	1	0	0	1	1	3349.9
0	0	1	0	0	1	0	1	1	3349.9
1	0	0	1	0	0	0	1	1	3350
0	0	1	0	0	0	0	1	0	3350.3
0	1	1	0	1	1	0	0	1	3350.7
1	1	0	1	1	0	0	0	1	3351.1
0	1	1	1	0	1	0	0	1	3351.3
0	0	0	1	1	1	0	0	1	3351.9
1	0	0	0	1	0	0	0	0	3352.3
1	1	0	0	0	1	0	1	1	3352.4
1	0	0	0	0	0	0	1	0	3352.6

0	1	0	1	1	1	0	0	1	3352.6
0	1	1	0	0	1	0	1	1	3352.7
1	1	0	1	0	0	0	1	1	3352.9
1	1	0	0	1	0	0	0	0	3353
0	1	1	0	0	0	0	1	0	3353.3
1	1	0	0	1	1	0	0	1	3353.3
0	0	0	1	1	0	0	0	0	3353.6
0	0	0	1	0	0	0	1	0	3353.8
0	0	0	0	0	1	0	1	0	3354.4
1	0	0	0	1	1	0	0	1	3354.6
1	1	0	0	0	0	0	1	0	3355.3
0	1	0	1	1	0	0	0	0	3355.3
0	1	0	1	0	0	0	1	0	3356.8
0	1	0	0	0	1	0	1	0	3357.3
1	0	1	0	0	0	0	0	1	3357.8
0	1	0	0	1	1	0	0	0	3357.9
0	0	1	1	1	0	0	0	1	3359.2
1	1	1	0	0	0	0	0	1	3360.3
0	1	1	1	1	0	0	0	1	3361.8
0	0	1	0	0	0	0	0	0	3361.9
0	0	1	1	0	0	0	1	1	3361.9
0	0	0	0	1	1	0	1	1	3362.2
0	0	1	0	1	0	0	1	1	3363.4
0	1	1	0	0	0	0	0	0	3364.2
1	0	0	1	0	0	0	0	1	3364.3
0	1	1	1	0	0	0	1	1	3365
0	1	0	0	1	1	0	1	1	3365
1	0	0	0	1	0	0	1	1	3365.1
1	1	0	1	0	0	0	0	1	3366
0	0	0	1	1	0	0	1	1	3366.1
0	0	1	0	0	1	0	0	1	3366.2
0	1	1	0	1	0	0	1	1	3366.3
1	0	0	0	0	0	0	0	0	3367.2
1	1	0	0	0	0	0	0	0	3367.7
1	1	0	0	1	0	0	1	1	3367.7
0	0	0	1	0	1	0	0	1	3368.3
0	1	1	0	0	1	0	0	1	3368.9
0	0	0	1	0	0	0	0	0	3369
0	1	0	1	1	0	0	1	1	3369
0	0	0	0	1	0	0	1	0	3369.4
0	1	0	1	0	0	0	0	0	3370.2
0	1	0	1	0	1	0	0	1	3370.3
1	0	0	0	0	1	0	0	1	3371.2

1	1	0	0	0	1	0	0	1	3371.8
0	1	0	0	1	0	0	1	0	3372.2
0	0	1	1	0	0	0	0	1	3374
0	0	1	0	1	0	0	0	1	3374.7
0	0	0	0	0	1	0	0	0	3375.4
0	1	0	0	0	1	0	0	0	3376.1
0	1	1	1	0	0	0	0	1	3376.5
0	1	1	0	1	0	0	0	1	3377.3
0	0	1	0	0	0	0	1	1	3378
1	0	0	0	1	0	0	0	1	3379.9
1	0	0	0	0	0	0	1	1	3380
1	1	0	0	1	0	0	0	1	3380.6
0	0	0	1	1	0	0	0	1	3380.7
0	1	1	0	0	0	0	1	1	3381
0	0	0	0	0	1	0	1	1	3381
0	0	0	1	0	0	0	1	1	3381.5
1	1	0	0	0	0	0	1	1	3382.6
0	1	0	1	1	0	0	0	1	3382.7
0	1	0	0	0	1	0	1	1	3384
0	0	0	0	0	0	0	1	0	3384.4
0	0	0	0	1	0	0	0	0	3384.4
0	1	0	1	0	0	0	1	1	3384.5
0	1	0	0	1	0	0	0	0	3384.9
0	1	0	0	1	1	0	0	1	3385.1
0	0	0	0	1	1	0	0	1	3386.6
0	1	0	0	0	0	0	1	0	3387.1
0	0	1	0	0	0	0	0	1	3389.3
0	1	1	0	0	0	0	0	1	3391.8
1	0	0	0	0	0	0	0	1	3394.8
1	1	0	0	0	0	0	0	1	3395.2
0	0	0	1	0	0	0	0	1	3396.1
0	0	0	0	1	0	0	1	1	3396.9
0	1	0	1	0	0	0	0	1	3397.7
0	1	0	0	1	0	0	1	1	3399.5
0	1	0	0	0	0	0	0	0	3399.6
0	0	0	0	0	1	0	0	1	3403.2
0	1	0	0	0	1	0	0	1	3403.6
0	0	0	0	0	0	0	1	1	3411.9
0	0	0	0	1	0	0	0	1	3412.1
0	1	0	0	1	0	0	0	1	3412.5
0	1	0	0	0	0	0	1	1	3414.4
0	0	0	0	0	0	0	0	1	3427
0	1	0	0	0	0	0	0	1	3427.1
