# Supplementary material for: Evaluating Methods for Isolating Total RNA and Predicting the Success of Sequencing Phylogenetically Diverse Plant Transcriptomes
Source: PLoS One. 2012 Nov 21;7(11):e50226. doi: 10.1371/journal.pone.0050226 (PMC3504007; doi:10.1371/journal.pone.0050226)
Supplement: Table S9 — The statistical fit of all possible linear combinations of factors in the large data set that excluded OD ratios. The fit of models was statistically compared using maximum likelihood statistics according to the Akaike information criterion (AIC). Models are arranged in the order of best-fitting models (lowest AIC) to poorer fitting models (higher AIC). Inclusion or absence of explanatory variables from models is shown by 1 and 0, respectively. (PDF) [file pone.0050226.s010.pdf]

Table S9 The statistical fit of all possible linear combinations of factors in the large data set that excluded OD ratios. The fit of models was statistically compared using maximum likelihood statistics according to the Akaike Information Criterion (AIC). Models are arranged in the order of the best-fitting models (lowest AIC) to poorer fitting models (higher AIC). The inclusion or absence of explanatory variables from models is shown by 1 and 0, respectively.

Tissue	RNA conc.	r26S:18S	RIN	Platform	µg RNA sequenced	Bases sequenced	AIC
1	0	1	1	1	1	0	9174
1	1	1	1	1	1	0	9177.3
1	0	0	1	1	1	0	9188.1
1	0	1	1	1	1	1	9189.9
1	1	0	1	1	1	0	9191.4
1	0	1	0	1	1	0	9193.1
1	1	1	1	1	1	1	9193.5
1	0	1	1	1	0	0	9193.7
1	1	1	1	1	0	0	9194.2
1	1	1	0	1	1	0	9196.5
1	0	0	1	1	1	1	9204.4
1	0	1	1	1	0	1	9206.8
1	1	0	1	1	1	1	9208
1	0	0	1	1	0	0	9208.4
1	1	0	1	1	0	0	9208.5
1	1	1	1	1	0	1	9209.7
1	0	1	0	1	1	1	9210.7
1	0	1	0	1	0	0	9213.2
1	1	1	0	1	0	0	9213.8
1	1	1	0	1	1	1	9214.3
1	0	0	0	1	1	0	9215.1
1	1	0	0	1	1	0	9218.2
1	0	0	1	1	0	1	9221.9
1	1	0	1	1	0	1	9224.6
1	0	1	0	1	0	1	9228.1
1	1	1	0	1	0	1	9231
1	0	0	0	1	1	1	9235
1	1	0	0	1	0	0	9236.6
1	0	0	0	1	0	0	9237.4
1	1	0	0	1	1	1	9238.6
1	0	0	0	1	0	1	9254.8
1	1	0	0	1	0	1	9256.6
1	0	1	1	0	1	0	9261.5
1	1	1	1	0	1	0	9263.7
1	0	1	1	0	1	1	9273

1	1	1	1	0	0	0	9276.3
1	1	1	1	0	1	1	9276.4
1	0	1	1	0	0	0	9277.2
1	0	0	1	0	1	0	9277.7
1	1	0	1	0	1	0	9279.5
0	0	1	1	1	1	0	9280.5
1	0	1	0	0	1	0	9280.8
1	1	1	0	0	1	0	9283
0	1	1	1	1	1	0	9284.1
1	0	1	1	0	0	1	9286.3
1	1	1	1	0	0	1	9288.6
1	0	0	1	0	1	1	9289.8
1	1	0	1	0	0	0	9292.4
1	1	0	1	0	1	1	9293.1
1	0	0	1	0	0	0	9294.1
1	0	1	0	0	1	1	9294.2
0	0	0	1	1	1	0	9294.6
1	1	1	0	0	0	0	9295.9
0	0	1	1	1	1	1	9296.3
1	0	1	0	0	0	0	9296.8
1	1	1	0	0	1	1	9297.6
0	1	0	1	1	1	0	9298.1
0	0	1	0	1	1	0	9299.8
0	1	1	1	1	1	1	9299.9
0	0	1	1	1	0	0	9302
0	1	1	1	1	0	0	9303.3
0	1	1	0	1	1	0	9303.4
1	0	0	1	0	0	1	9303.8
1	1	0	1	0	0	1	9305.5
1	0	1	0	0	0	1	9307.9
1	0	0	0	0	1	0	9308.9
1	1	1	0	0	0	1	9310.1
1	1	0	0	0	1	0	9310.3
0	0	0	1	1	1	1	9311.3
0	0	1	1	1	0	1	9314.5
0	1	0	1	1	1	1	9315
0	0	0	1	1	0	0	9316.5
0	0	1	0	1	1	1	9317.1
0	1	0	1	1	0	0	9317.5
0	1	1	1	1	0	1	9317.7
0	1	1	0	1	1	1	9320.6
0	0	1	0	1	0	0	9321.8
0	0	0	0	1	1	0	9322.6

0	1	1	0	1	0	0	9323.3
1	1	0	0	0	0	0	9324.1
1	0	0	0	0	1	1	9325.5
0	1	0	0	1	1	0	9326
1	0	0	0	0	0	0	9327.1
1	1	0	0	0	1	1	9328.4
0	0	0	1	1	0	1	9330.1
0	1	0	1	1	0	1	9333.1
0	0	1	0	1	0	1	9335.9
0	1	1	0	1	0	1	9339.2
1	0	0	0	0	0	1	9341.5
1	1	0	0	0	0	1	9341.9
0	0	0	0	1	1	1	9343.7
0	0	0	0	1	0	0	9346.5
0	1	0	0	1	0	0	9346.8
0	1	0	0	1	1	1	9347.3
0	0	0	0	1	0	1	9365
0	1	0	0	1	0	1	9367.2
0	0	1	1	0	1	0	9382.5
0	1	1	1	0	1	0	9385.1
0	0	1	1	0	1	1	9388
0	1	1	1	0	1	1	9391.6
0	0	1	1	0	0	0	9397
0	1	1	1	0	0	0	9397.3
0	0	0	1	0	1	0	9398.1
0	0	1	1	0	0	1	9400
0	1	0	1	0	1	0	9400.4
0	1	1	1	0	0	1	9403.1
0	0	0	1	0	1	1	9405.1
0	0	1	0	0	1	0	9406.4
0	1	0	1	0	1	1	9408.6
0	1	1	0	0	1	0	9409.1
0	1	0	1	0	0	0	9412.7
0	0	0	1	0	0	0	9412.9
0	0	1	0	0	1	1	9413.7
0	1	1	0	0	1	1	9417.2
0	0	0	1	0	0	1	9417.6
0	1	0	1	0	0	1	9420.2
0	0	1	0	0	0	0	9421
0	1	1	0	0	0	0	9421.6
0	0	1	0	0	0	1	9425.9
0	1	1	0	0	0	1	9429
0	0	0	0	0	1	0	9438.1

0	1	0	0	0	1	0	9439.9
0	0	0	0	0	1	1	9451.5
0	1	0	0	0	0	0	9452.9
0	1	0	0	0	1	1	9454.6
0	0	0	0	0	0	1	9465.6
0	1	0	0	0	0	1	9467
