# Supplementary material for: Evaluating Methods for Isolating Total RNA and Predicting the Success of Sequencing Phylogenetically Diverse Plant Transcriptomes
Source: PLoS One. 2012 Nov 21;7(11):e50226. doi: 10.1371/journal.pone.0050226 (PMC3504007; doi:10.1371/journal.pone.0050226)
Supplement: Appendix S1 — Eighteen protocols used to isolate total RNA from plant tissue included in this study. (PDF) [file pone.0050226.s011.pdf]

Appendix S1 Eighteen protocols used to isolate total RNA from plant tissue included in this study.

Methods for RNA isolation

We attempted to isolate RNA from 1115 plant samples, represented by 695 species of vascular and non-vascular plants. RNA isolation was achieved using 13 distinct protocols. Many of the protocols share elements or combine components from several methods. For each method, we describe the reagents and procedures used, and identify the researchers or institute that implemented the protocol.

Due to the potential for contamination and degradation by RNase enzymes, as well as health concerns in handling some substances and chemicals, best practices in aseptic wet lab techniques must be practiced at all times during RNA isolation. Chief among these are the critical need to avoid contamination of samples by using extreme care when moving liquids and opening and closing tubes to avoid aerosols. Because of the risk of degradation by RNase enzymes, it is essential to use sterile RNase-free equipment, disposable plastics and solutions. RNase degradation and contamination can be avoided by keeping samples constantly frozen at low temperature (< -80°C) prior to adding buffers that denature or immobilize RNase. Treating equipment with RNase denaturants (e.g. RNase Zap, Ambion, Austin, TX) and solutions with diethylpyrocarbonate (DEPC) can also prevent contamination and/or degradation of samples, but it can have some negative effects on samples[1]. Many additional helpful tips for successful RNA isolation are available in Sambrook and Russell[1] and in Appendix A of Qiagen's RNeasy Mini Handbook downloadable from www.qiagen.com.

Standard equipment used

Although the reagents vary among protocols, most methods used the same basic equipment, which include:

- Ceramic or porcelain mortar and pestle for tissue homogenization, or some other equipment that can homogenize frozen tissue (e.g. bead mill)
- Water bath or heating block, capable of holding temperatures up to 70°C
- Non-refrigerated microcentrifuge (with rotor for 2 ml tubes)
- Refrigerated microcentrifuge (with rotor for 2 ml tubes) – only for protocols where noted
- Liquid nitrogen (and associated thermos or dewer)
- Sterile, RNase-free disposable tips with filter barriers
- Sterile, RNase-free disposable microcentrifuge tubes (2 ml and 15 ml were most commonly used, but sizes vary among protocols)
- Pipettors – (capable of pipetting various volumes between 0.1-1000μl)
- Stainless steel spatulas
- RNase-free water
- Sterile razor blades
- Analytical balance
- Glass beakers, flasks and graduated cylinders

Protocol 1: Qiagen RNeasy Plant Minikit

Implemented by: Jim Leebens-Mack and Charlotte Carrigan

The first protocol used for isolation of total RNA was Qiagen's RNeasy Plant Minikit (Qiagen, Valencia, CA). These kits are commercially available and widely used in RNA isolation. For detailed methods associated with this kit we refer readers to the "RNeasy Mini Handbook" available for download at www.qiagen.com.

Protocol 2: McKenzie et al's Qiagen hybrid method

Implemented by: Jim Leebens-Mack and Charlotte Carrigan

This protocol was developed by McKenzie et al[2] to facilitate the isolation of RNA from woody plants rich in phenolics and polysaccharides, such as grapes (Vitaceae) and fruit bearing Rosaceae (apples, cherries and pears). The protocol is provided on Qiagen's website as an alternative method to be used in combination with their RNeasy Plant Minikit (see www.qiagen.com/products/rnastabilizationpurification/rneasysystem/rneasyplantmini.aspx#Tabs=t2, under the "Protocols" tab). We repeat the protocol here in the event that this protocol is removed from Qiagen's website or readers find it difficult to obtain the original publication.

<u>Reagents</u>
Lysis Buffer:

- 4 M guanidine isothiocyanate
- 0.2 M sodium acetate, pH 5.0
- 25 mM EDTA
- 2.5% (w/v) PVP -40 (polyvinylpyrollidone, average molecular weight, 40,000)
- 1% (v/v) β-mercaptoethanol (β-ME); add immediately before use.

Other reagents:

- 20% (w/v) sarkosyl
- Ethanol (96-100%)

<u>Procedure</u>

1. Grind sample (up to 50 mg) in liquid nitrogen to a fine powder using a mortar and pestle. Transfer the tissue powder and liquid nitrogen to an appropriately sized tube, and allow the liquid nitrogen to evaporate. Do not allow the sample to thaw. Continue immediately with step 2

   Note: Incomplete grinding of the starting material will lead to reduced RNA yields.

2. Add 600 µl lysis buffer to a maximum of 50 mg of tissue powder. Vortex vigorously.
3. Add 60 µl of 20% sarkosyl. Incubate at 70°C in a water bath or heating block for 10 min with vigorous shaking or intermittent vortexing.

Note: For samples with a high starch content, incubation at elevated temperatures should be omitted to prevent swelling of the starting material.

4. Pipet the lysate directly onto a QIAshredder Spin Column (lilac-colored column, supplied in the RNeasy Plant Mini Kit) placed in a 2 ml collection tube. Centrifuge for 2 min at maximum speed (>14,000 *g* [rcf]).
5. Carefully transfer the supernatant of the flow-through fraction to a new microcentrifuge tube without disturbing the cell-debris pellet in the collection tube.

   Note: It may be necessary to cut the end off the pipet tip in order to pipet the lysate onto the QIAshredder Spin Column. Centrifugation through the QIAshredder Spin Column removes cell debris and simultaneously homogenizes the lysate. While most of the cell debris is retained on the QIAshredder Spin Column, a very small amount of cell debris will pass through and form a pellet in the collection tube. Be careful not to disturb this pellet while transferring the lysate to a new microcentrifuge tube.
6. Add 0.5 volumes (usually 300 µl) ethanol (96–100%), and mix well by pipetting. If some lysate is lost during homogenization (step 4), reduce volume of ethanol proportionally. A precipitate may form after the addition of ethanol, but this will not affect the RNeasy procedure.
7. Continue with step 6 of the RNeasy Mini Protocol for Isolation of Total RNA from Plant Cells and Tissues and Filamentous Fungi in the RNeasy Mini Handbook.

Protocol 3: CTAB-PVP Method

Implemented by: Beijing Genomics Institute

Note: a similar protocol was used by C. dePamphilis and P. Ralph

<u>Reagents:</u>
CTAB-PVP Buffer:

- CTAB (Hexadecyltrimethylammonium bromide; 2% w/v)
- PVP-40 (2% w/v)
- 100mM Tris-HCl (pH8.0)
- 25 mM EDTA
- 2 M NaCl (Warmed to 65℃ in a water bath to suspend in solution)
- Add β-ME to final concentration of 2% before use

SSTE buffer:

- 1 M NaCl
- SDS (0.5% w/v)
- 10mM Tris-HCl (pH8.0)
- EDTA (1 mM)

Other reagents:

- 75% ethanol (treated with 0.1% DEPC)

- 96-100% ethanol
- Acid phenol (pH4.5)
- Chloroform
- Isoamyl alcohol
- 10 M LiCl
- Glycogen (5mg/ml)
- 3M Sodium Acetate (pH5.2)
- RNase free water

Procedure

1. Grind tissue to a powder in liquid nitrogen.
2. Add 200-500 mg of ground, frozen tissue to 3.0 ml of pre-heated extraction buffer in a 5 ml tube.
3. Vortex the tube until the tissue is mixed with the buffer.
4. Incubate the tube at 65°C for 30 min. (min), vortexing briefly (15 seconds) every 2-3 min during the incubation.
5. Aliquot the mixture into four 2 ml-RNase free tubes, 1 ml each tube.
6. Spin the tube at 12,000g for 10 min in a microcentrifuge. All of the insoluble matter should form a pellet at the bottom of the tube.

   Note: When used for algae the centrifugation was performed at 3,000g
7. Pour the supernatant into a new 2 ml RNase free tube.
   Note: When used for algae, 15 ml tubes were used.
8. Add equal volume of 24:1 chloroform:isoamyl alcohol to fill the tube.
9. Vortex tubes until the phases mix and appear cloudy, then incubate at 20-25°C for 5 min..
10. Spin the tubes at 12,000g for 10 min in a microcentrifuge.
    Note: When used for algae the centrifugation was performed at 3,000g
11. Transfer the upper, aqueous phase to new 2 ml RNase free tubes, repeat step 7 to 9 one more time.
    Note: When used for algae, 15 ml tubes were used.
12. Transfer the upper, aqueous phase to new 2 ml RNase free tubes, add 1/3 volume of 10M LiCl to each tube, mix and let stand at 4°C for 6-8 hrs or overnight to precipitate RNA.
13. Spin the tubes at 18,000 g for 20 min in a microcentrifuge and decant the supernatant, taking care not to lose the pellet
14. Add 1 ml 75% ethanol to the pellet.
15. Spin the tube at maximum speed (>11,270 *g*) for 5 min in a microcentrifuge, decant the supernatant carefully.
16. Repeat steps 14 and 15 one more time.
17. Open cap and air-dry the pellet.

18. Add 30µl RNase free water to dissolve the pellet, and then add 70 µl SSTE buffer to each tube.
19. Combine all 4 tubes into 1 tube.
20. Add equal volume of 25:24:1 acid phenol:chloroform:isoamyl alcohol to the tube.
21. Vortexes the tubes until the phases mix and appear cloudy, then incubate at 20°C for 5 min..
22. Spin the tube at 12,000g for 10 min.
23. Transfer the upper, aqueous phase to a new 2 ml RNase free tube and add equal volume of 24:1 chloroform:isoamyl alcohol to the tube.
24. Vortex the tubes until the phases mix and appear cloudy, then incubate at 20°C for 5 min..
25. Spin the tube at 12,000g for 10min in a microcentrifuge.
26. Transfer the upper, aqueous phase to a new 2 ml RNase free tube, add 2 volumes of cooled 100% ethanol, 1/10 volumes of NaAc (pH5.2) and 2 µl glycogen, mix and incubate at -20°C for 2 hrs.
27. Spin the tube at 18,000 g for 20 min in a microcentrifuge, and then decant the supernatant, taking care not to lose the pellet
28. Add 1 ml 75% cooled ethanol to the pellet and leave at 20°C for 3 min..
29. Centrifuge at 4°C for 5 min at 12,000g. Decant the liquid carefully, taking care not to lose the pellet. Briefly centrifuge to collect the residual liquid and remove it with a pipette.
30. Wash the pellet twice with cooled 75% DEPC-ethanol, open cap and air-dry the pellet.
31. Add 30µl RNase-free water to dissolve the pellet.
32. Treat RNA with DNase I as per supplier's protocols.

Protocol 4: CTAB-PVP-TRIzol Method

Implemented by: Beijing Genomics Institute

<u>Reagents</u>
CTAB-PVP buffer:
- CTAB (2% w/v)
- PVP-40 (2% w/v)
- 100mM Tris-HCl (pH8.0)
- 25mM EDTA
- 2 M NaCl
- Spermide (0.5g/L),

(Warmed to 65°C in a water bath to suspend in solution)
- Add β-ME to final concentration of 2% before use

SSTE buffer:

- 1 M NaCl
- SDS (0.5% w/v)
- 10mM Tris-HCl (pH8.0)
- 1mM EDTA

Other Reagents:

- 75% ethanol (DEPC treated)
- 100% ethanol
- Acid phenol (pH4.5)
- Chloroform
- Isoamyl alcohol
- 10M LiCl
- Glycogen (5mg/ml)
- 3M NaAc pH5.2
- TRIzol reagent (Invitrogen)
- RNase free water

Procedure

1. Grind tissue to a powder in liquid nitrogen.
2. Add 200-500 mg of ground tissue to 3.0 ml of pre-heated extraction buffer in a 5 ml tube.
3. Vortex the tube until the tissue is mixed with the buffer.
4. Incubate the tube at 65℃ for 30 min., vortexing briefly (15 seconds) every 2-3 min during the incubation.
5. Aliquot the mixture into four 2 ml RNase free tubes, 1 ml in each tube.
6. Spin the tube at 12,000g for 10 min in a centrifuge. All of the insoluble matter should form a pellet at the bottom of the tube.
7. Pour the supernatant into a new 2 ml tube.
8. Add an equal volume of 24:1 chloroform:isoamyl alcohol to fill the tube.
9. Vortex tubes until the phases mix and appear cloudy; incubate at 20°C for 5 min..
10. Spin the tubes at 12,000g for 10 min in a centrifuge.
11. Transfer the upper aqueous phase to new 2 ml RNase free tubes, repeat steps 8 to 10 one more time.
12. Transfer the upper, aqueous phase to new 2 ml RNase free tubes, add 1/3 volume of 10M LiCl to each tube, mix and let stand at 4℃ for 6-8 hrs or overnight to precipitate RNA.
13. Spin tubes at 18,000g for 20 min in a centrifuge and decant the supernatant, taking care not to lose the pellet.
14. Add 1 ml 75% cooled ethanol to the pellet.

15. Spin the tube at maximum speed for 5 min in a centrifuge, decant the supernatant carefully. Repeat steps 14 and 15 one more time.
16. Open cap and air-dry the pellet.
17. Add 30µl RNase free water to dissolve the pellet, and then add 300µl TRIzol reagent and equal volume of chloroform to TRIzol reagent (Invitrogen). Vortex vigorously and store at 20°C for 5 min.
18. Centrifuge at >12,000g for 10 min..
19. Transfer the upper, aqueous phase to a new 2 ml RNase free tube, add 2 volumes of cooled 100% ethanol, 1/10 volume of NaAc and 2µl of glycogen. Mix and incubate at -----20℃ for 2 hrs.
20. Spin tubes at >12,000g for 20 min at 4℃ in a centrifuge
21. Decant the supernatant taking care not to lose the pellet and add 1 ml 75% ethanol to the pellet. Let tube stand at 20°C for 3 min..
22. Centrifuge at 4°C for 5 min at >12,000g. Decant the liquid carefully, taking care not to lose the pellet. Briefly centrifuge to collect the residual liquid and remove it with a pipette.
23. Repeat steps 21 and 22 one more time.
24. Open cap and air dry the pellet.
25. Add 10-30µl RNase free water to dissolve the pellet.

Protocol 5: pBIOZOL Method

Implemented by: Beijing Genomics Institute

<u>Reagents</u>

- 5M NaCl
- Chloroform
- Isopropyl alcohol
- 75% ethanol (DEPC treated)
- pBIOZOL Reagent (Beijing Bai billion New Technology Co., Beijing, China)
- RNase-free water

<u>Procedure</u>

1. Grind tissue to a powder in liquid nitrogen.
2. Add 1.3 ml of cold (4°C) pBIOZOL Reagent for up to 100 mg of frozen, ground tissue. Mix by briefly vortexing or flicking the bottom of the tube until the sample is thoroughly suspended.
3. Incubate the tube for 5 min at 20°C.

   Note: Lay the tube down horizontally to maximize surface area during RNA extraction.
4. Centrifuge for 2 min. at 12,000g. Transfer the supernatant to an RNase-free tube.

5. Add 100µlof 5M NaCl to the extract and tap tube to mix.
6. Add 300µl of chloroform. Mix thoroughly by inversion.
7. Centrifuge the sample at 4°C for 10 min. at 12,000g to separate the phases. Transfer the top aqueous phase to an RNase-free tube.
8. Add to the aqueous phase an equal volume of isopropyl alcohol. Mix and let stand at 20°C for 10 min.
9. Centrifuge the sample at 4°C for 10 min. at 12,000g.
10. Decant the supernatant, taking care not to lose the pellet and add 1 ml of chilled 75% ethanol to the pellet.

    Note: Pellet may be difficult to see.
11. Centrifuge at room temperature for 5 min at 12,000g. Decant the liquid carefully, taking care not to lose the pellet. Briefly centrifuge to collect the residual liquid and remove it with a pipette.
12. Add 10-30 µl RNase-free water to dissolve the RNA. Pipette the water up and down over the pellet to dissolve the RNA. If any cloudiness is observed, centrifuge the solution at room temperature for 1 min at 12,000 × g and transfer the supernatant to a fresh tube.

Protocol 6: pBIOZOL and Qiagen RNeasy Plant Mini Kit Method

Implemented by: Beijing Genomics Institute

Reagents
Acid phenol (pH 4.5)
Chloroform
Isopropyl alcohol
75% ethanol (DEPC treated)
100% ethanol
5M NaCl
pBIOZOL Reagent (Beijing Bai billion New Technology Co., Beijing, China)
RNeasy Plant Mini Kit (Qiagen)
RNase-free water

Procedure
1. Grind tissue to a powder in liquid nitrogen.
2. Add 1.3 ml of cold (4°C) pBIOZOL reagent for up to 100mg of frozen ground tissue. Mix by briefly vortexing or flicking the bottom of the tube until the sample is thoroughly re-suspended.
3. Incubate the tube for 5 min. at room temperature.

    Note: Lay the tube down horizontally to maximize surface area during RNA extraction.

4. Centrifuge for 10 min. at 12,000g in a microcentrifuge at room temperature. Transfer the supernatant to a new 1.5 ml RNase-free tube.

5. Add 100µl of 5M NaCl and 300µl chloroform, vortex vigorously.
4. Centrifuge at 12,000g for 10 min.
5. Transfer the top aqueous phase to a new 1.5 ml RNase-free tube, add an equal volume of 5:1 acid phenol:chloroform to the tube.
6. Vortex the tube until the phases mix and appear cloudy, then incubate at 20°C for 5 min..
7. Centrifuge at 12,000g for 10 min.
8. Transfer the top aqueous phase to a new 1.5 ml RNase-free tube, add to the aqueous phase equal volume of 24:1 chloroform:isoamyl alcohol. Vortex the tube until the phases mix and appear cloudy, then incubate at room temperature for 5 min..
9. Centrifuge at 12,000g for 10 min.
10. Transfer the top aqueous phase to a new 1.5 ml RNase-free tube, add 1/2 volume of 100% ethanol.
11. Pour the contents of the tube into a Qiagen mini RNA spin column (pink), until the column is almost filled with liquid.
12. Cap the tube and centrifuge at 12,000g for 15 sec. The column should be empty at the end of this spin.
13. Discard the flow-through from the collection tube.
14. Repeat the previous two steps with the same mini RNA spin column, until all of the liquid in the tube(s) has been passed through the column. The nucleic acid is now bound to the silica membrane in the spin column.
15. Apply 700 µl of solution RW1 to the spin column.
16. Cap the tube and centrifuge at 12,000g for 15 seconds. The column should be empty at the end of this spin.
17. Discard the flow-through from the collection tube.
18. Apply 500 µl of solution RPE to the spin column. Cap the tube and centrifuge at 12,000g for 15 seconds at 12, 000 g. The column should be empty at the end of this spin.
19. Discard the flow through.
20. Repeats previous two steps one time.
21. Spin at maximum speed for 2 min. to remove remaining liquid from the silica membrane.
22. Transfer the spin column to a new 1.5 ml conical bottom microcentrifuge tube
23. Add 30-50 µl of RNase-free water to the column, and then let tube incubate at 20°C for 3 min..
24. Spin at maximum speed for 1 min to collect RNA solution.

Protocol 7: pBIOZOL-LiCl Method

Implemented by: Beijing Genomics Institute

Reagents

- Acid phenol (pH 4.5)
- Chloroform
- Isopropyl alcohol
- 75% ethanol (DEPC treated)
- 100% ethanol
- 2M NaAc (pH 4.2)
- 3M NaAc (pH5.2)
- 5M NaCl
- 10M LiCl
- pBIOZOL Reagent (Beijing Bai billion New Technology Co., Beijing, China)
- RNase-free water

SSTE Buffer:

- 1M NaCl
- SDS (0.5% w/v)
- 10mM Tris-HCl (pH8.0)
- 1 mM EDTA

Procedure

1. Grind tissue to a powder in liquid nitrogen.
2. Add 1.3 ml of cold (4°C) pBIOZOL reagent for up to 100mg gram of frozen, ground tissue. Mix by briefly vortexing or flicking the bottom of the tube until the sample is thoroughly suspended.
3. Incubate the tube for 5 min. at room temperature.
   Note: Lay the tube down horizontally to maximize surface area during RNA extraction.
4. Centrifuge at 12,000g for 2 min..
5. Transfer the supernatant to a new 1.5 ml RNase-free tube.
6. Add 50 µl of 2M NaAc (pH4.2) to the extract and tap tube to mix, and then add 100ul 5M NaCl and 300µl chloroform, vortex vigorously.
7. Centrifuge the mixture at 4°C for 10 min. at 12,000g to separate the phases. Transfer the top aqueous phase (about 400-500 µl) to a new 1.5 ml RNase-free tube.
8. Add to the aqueous phase 1/3 volume of 10M LiCl. Mix and let stand at 4°C overnight.
9. Centrifuge the mixture at 4°C for 20 min. at >12,000g.
10. Decant the supernatant, taking care not to lose the pellet and add 1 ml of 75% ethanol to the pellet and stand the tube at room temperature for 3 min..
    Note: Pellet may be difficult to see.
11. Centrifuge at 4°C for 3 min at >12,000g. Decant the liquid carefully, taking care not to lose the pellet. Briefly centrifuge to collect the residual liquid and remove it with a pipette.
12. Repeat the previous two steps.

13. Add 50 µl RNase-free water to dissolve the RNA pellet. Pipette the water up and down over the pellet to dissolve the RNA.
    Note: If you extract more than 100mg plant tissues, combine different extractions to one tube.
14. Add SSTE buffer to RNA to a total volume of 600µL, then add equal volume of 25:24:1 acid phenol:chloroform:isoamyl alcohol to the tube.
15. Vortex the tube until the phases mix and appears cloudy, then incubate at 20°C for 5 min..
16. Centrifuge at 12,000g for 10 min in a microcentrifuge.
17. Transfer the top, aqueous phase to a new 1.5 ml RNase-free tube, add equal volume of 24:1 chloroform:isoamyl alcohol to the tube.
18. Vortex the tube until the phases mix and appear cloudy, then incubate at 20°C for 5 min.
19. Centrifuge at 12,000g for 10 min.
20. Transfer the top aqueous phase to a new 1.5 ml RNase-free tube, add to the aqueous phase 2 volumes of 100% ethanol, 1/10 volume of 3M NaAc (pH 5.2) and 2µl 5mg/ml glycogen. Invert tube to mix and store at -20°C for 2 hours.
21. Centrifuge at 4°C for 20 min. at >12,000g, decant the supernatant carefully to avoid losing the pellet.
22. Add 1 ml of 75% ethanol to the pellet and incubate at 20°C for 3 min..
23. Centrifuge at 4°C for 5 min at 12,000 *g*. Decant the liquid carefully, taking care not to lose the pellet. Briefly centrifuge to collect the residual liquid and remove it with a pipette.
24. Repeat step 18 and 19.
25. Open cap and air-dry the pellet no more than 5 min..
26. Add 30µl RNase-free water to dissolve the pellet.
27. Before library construction, treat RNA with DNase I.

Protocol 8: CTAB/Acid Phenol/Silica Membrane Method

Implemented by: Michael Deyholos

This protocol combines elements of standard CTAB, acid phenol, and silica-membrane protocols. The protocol was developed to extract total RNA from a wide range of plant species and tissues, and to do so in the smallest volume possible to yield >20µg of total RNA using as little as 50mg of fresh tissue. Maintaining a small extraction volume also allows for many samples to be processed in parallel in microcentrifuge tubes; at least 12 samples can be processed in 3-4 hours. The protocol has been used successfully with dozens of species, including tissues rich in polysaccharides and/or secondary metabolites. There are four organic extractions in total (three chloroform and one phenol:chloroform). For many species/tissues, the full set of chloroform extractions in the order specified is required to maximize RNA purity and to prevent phase inversion during the acid phenol extraction.

Because the RNA is protected from RNase by denaturants throughout most of the protocol, it is not necessary to use specially treated (e.g. baked or DEPC) labware or solutions. We have also found that QIAshredder columns have little positive or negative impact on quality and yields, although they may be useful for some tissues that are not easily disrupted by grinding in a mortar.

Instead of using a Qiagen silica membrane spin column (also available from other manufacturers), it is also possible to precipitate the RNA after the last organic extraction (step 22). However, the silica membrane columns provide more reliable recovery of RNA (especially in a high-throughput, service environment), and allow for the convenient removal of residual DNA through an on-column digestion. We have not found it beneficial to collect more than one RNA elution from a spin column, or to pass the eluate through the same column twice.

The binding capacity of the Qiagen column (~100µg RNA) exceeds the yield of RNA that can be extracted from tissue in a single 2 ml microcentrifuge tube. Therefore, when yields of >20µg total RNA / ~1 g fresh weight of tissue are required, it is most efficient to aliquot the tissue sample between two tubes, process the aliquots independently through stage 24, then pool the samples into a single column.

Both the acid phenol extraction and the QIAgen silica membrane washes are biased in favour of the recovery of RNA over DNA. Indeed, there appears to be little residual DNA present even before on-column DNase I digestion. Nevertheless, it is probably worthwhile to conduct the digestion on all samples to limit the possibility that any genomic DNA molecules could be used as a sequencing template.

<u>Reagents</u>

CTAB extraction buffer (for 200 ml final volume):

- 40 ml Tris-HCl pH 7.5
- 10 ml 0.5M EDTA
- 35.04g NaCl
- 4g CTAB
- 4g SDS (sodium dodecyl sulfate)
- 4g PVP (polyvinylpyrrolidone)
- 8 ml β-ME (2-mercaptoethanol)

Note: Heat to 65°C to dissolve components in solution. SDS may not completely dissolve)

Other reagents:

- Saturated NaOH solution
- Chloroform:Isoamyl Alcohol (24:1)
- Phenol:Chloroform (5:1, pH4.5).

Note: It is essential to use acid-equilibrated phenol, rather than Tris-buffered phenol.

- Qiagen’s RLT, RW1, RPE, DNase digestion solutions, plus Plant minikit spin columns (pink)

Mortars should be rinsed with saturated NaOH to remove residual RNA, and then rinsed with DEPC-treated water.

<u>Procedure</u>

1. Grind tissue to a powder in liquid nitrogen.
2. Add 400-600mg of ground, frozen tissue to 1.4 ml of pre-heated extraction buffer in a 2 ml microcentrifuge tube.
3. Vortex the tube until the tissue is mixed with the buffer. To facilitate mixing, you may have to invert the tube on the vortex, and/or heat it briefly in a 65°C water bath.
4. Incubate the tube at 65°C for 10-15 min., vortexing briefly (15 seconds) twice during the incubation.
5. Spin the tube at maximum speed (>11,269 *g*) for 3 min in a microcentrifuge. All of the insoluble matter should form a pellet at the bottom of the tube.
6. Pour the supernatant into a new 2 ml tube.
7. Solvent Extraction #1: Add enough 24:1 chloroform:isoamyl alcohol to fill the tube.
8. Vortex the tube for 15 seconds or until the phases mix and appear cloudy.
9. Spin the tube at maximum speed (>11,269 *g*) for 3 min in a microcentrifuge. Most of the chlorophyll will be dissolved in the lower, organic phase.
10. Transfer the upper, aqueous phase to a new 2 ml tube, using a disposable pipette. Avoid transferring any of the material (usually a white precipitate) from the boundary between the phases.
11. Solvent Extraction #2: Add 24:1 chloroform:isoamyl alcohol to the tube containing the aqueous phase (this should be at least 900µl of 24:1 chloroform:isoamyl).
12. Vortex the tube for 15 seconds or until the phases mix and appear cloudy.
13. Spin the tube at maximum speed (>11,269 *g*) for 3 min in a microcentrifuge.
14. Transfer the upper, aqueous phase to a new 2 ml tube, using a disposable pipette. Avoid transferring any of the material (usually a white precipitate) from the boundary between the phases.
15. Solvent Extraction #3: Add 1 ml 5:1 phenol:chlorofom pH4.5 to the tube containing the aqueous phase.
16. Vortex the tube for 15 seconds or until the phases mix and appear cloudy.
17. Spin the tube at maximum speed (>11,269 *g*) for 3 min in a microcentrifuge.
18. Transfer the upper, aqueous phase to a new 2 ml tube, using a disposable pipette. Avoid transferring any of the material (usually a white precipitate) from the boundary between the phases.
19. Solvent Extraction #4: Add 24:1 chloroform:isoamyl alcohol to the tube containing the aqueous phase (this should be at least 900µl of 24:1 chloroform:isoamyl).
20. Vortex the tube for 15 seconds or until the phases mix and appear cloudy.

21. Spin the tube at maximum speed (>11,269 *g*) for 3 min in a microcentrifuge.
22. Transfer the upper, aqueous phase to a new 2 ml tube, using a disposable pipette. Avoid transferring any of the material (usually a white precipitate) from the boundary between the phases.
23. Estimate the volume of the aqueous phase based on the markings on the tube. Add at least 0.5 volumes of solution RLT, and mix by briefly shaking.
24. Estimate the new total volume in the tube, and add 0.5 volumes of 95-100% ethanol. Mix by briefly shaking.
25. Pour the contents of the tube into a Qiagen miniRNA spin column (pink), until the column is almost filled with liquid.
26. Cap the tube and spin for 15 seconds at >5,000 *g*. The column should be empty at the end of this spin.
27. Discard the flow-through from the collection tube.
28. Repeat the previous two steps with the same miniRNA spin column, until all of the liquid in the tube(s) has been passed through the column. The nucleic acid is now bound to the silica membrane in the spin column.
29. Apply 350µl of solution RW1 to the spin column.
30. Cap the tube and spin for 15 seconds at >5,000 *g*. The column should be empty at the end of this spin.
31. Discard the flow-through from the collection tube.
32. Apply 80µl of DNase digestion solution to the membrane of the spin column.
33. Incubate at room temperature for 15 min.
34. Apply 350µl of solution RW1 to the spin column.
35. Cap the tube and spin for 15 seconds at >5,000 *g*. The column should be empty at the end of this spin.
36. Discard the flow-through from the collection tube.
37. Apply 500µl of solution RPE to the spin column.
38. Cap the tube and spin for 15 seconds at >5,000 *g*. The column should be empty at the end of this spin.
39. Discard the flow-through from the collection tube.
40. Apply 500µl of solution RPE to the spin column.
41. Cap the tube and spin for 15 seconds at >5,000 *g*. The column should be empty at the end of this spin.
42. Discard the flow-through from the collection tube.
43. Transfer the spin column to a new collection tube, and spin at maximum speed for 3 min. to remove remaining liquid from the silica membrane.

44. Transfer the spin column to a new 1.5 ml conical bottom microcentrifuge tube
45. Add 44µl of RNase-free water to the column.
46. Spin at maximum speed for 1 min to elute.

Protocol 9: CTAB/Acid Phenol/Silica Membrane Method

Implemented by: Henrietta Myburg and Marc Johnson

This protocol is a modification of protocol 8. It was developed after protocol 8 and several commercially available plant RNA isolation kits failed to produce a sufficient yield and quality of RNA from *Oenothera* spp. (Onagraceae) for next-generation sequencing. *Oenothera* are rich in polysaccharides, oils, flavonoids and complex ellagitannins that likely interfere with isolation. We suspect that this protocol will be most useful for species and tissues with complex secondary chemistry and rich in oils (e.g. some Rosaceae and Pinaceae). The most important modifications to this protocol versus Protocol 8 is the use of less plant tissue, more extraction buffer, and repeating solvent extractions until the interphase is clean of debris. The protocol is regrettably longer and more involved than Protocol 8. We attempted to remove or reduce the replication of the solvent extractions steps without success (i.e. yield and quality are always decreased in *Oenothera* when any steps are removed).

Reagents

The solutions are identical to Protocol 8 except we added 2µl of β-ME to 1 ml of CTAB extraction buffer (instead of 40µl/1ml) immediately prior to the RNA extraction and deleted the addition of SDS. We also wiped the bench, mortars, pestles with RNaseZap Solution (Ambion, Austin, TX) immediately prior to grinding tissue and rinsed the mortar and pestles with ddH_20.

Procedure

1. Chill mortar and pestle with liquid nitrogen (fill the mortar carefully with liquid nitrogen, let it evaporate, repeat one more time. Keep the pestle in the mortar when doing this.)
2. Fill the mortar again; add 50-100mg of tissue into the mortar while the liquid nitrogen is still “bubbling”.
3. Carefully grind tissue to a powder in liquid Nitrogen. (NOTE: Add more nitrogen if needed, but it is important to be careful when you pour the liquid nitrogen. Especially if you are grinding a small amount of tissue in a small sized mortar. The liquid nitrogen tends to “blow” the tissue out of the mortar.)
4. Add 2 ml to 5 ml (depending on the amount of tissue you start with) of prepared extraction buffer on top of the tissue powder and mix in with the pestle. The extraction buffer will freeze because the mortar is still very cold from the liquid NITROGEN. Do not worry about this – start grinding your next sample. The tissue/CTAB mix will slowly thaw and at some point you will be able to mix it well using the pestle.

Note: Keep in mind that if you use more extraction buffer you will be working in more 2 ml tubes per sample and that the number of tubes per sample will increase once you reach the Qiagen RNeasy part of the protocol. Thus, there will be a lot of pipetting into the Qiagen spin column. One can do the solvent extraction steps in 15 ml Falcon tubes, but keep in mind that you will still have to pass a significantly larger amount of liquid through the Qiagen spin column.

5. Once thawed and well mixed, pipette or pour the slurry into a 2 ml tube. If the slurry is too thick, cut the tip of the 1 ml pipette tip with a clean razor blade or scissors. If you start with a lot of tissue, you might have to rinse the side of the mortar with an additional 1 ml of extraction buffer (or more if needed.)
6. Vortex each tube until the tissue is mixed with the buffer. To facilitate mixing, you may have to invert the tube on the vortex, and/or heat it briefly in a 65°C water bath.
7. Incubate tubes at 65°C for 10-15 min., vortexing briefly (15 seconds) twice during the incubation.
8. Spin the tubes at maximum speed (>14,000 *g*) for 3 min in a microcentrifuge. All of the insoluble matter should form a pellet at the bottom of the tube.
9. Pour or pipette the supernatant into a new 2 ml tube. Make sure that the total volume of the supernatant per tube is ≤ 1 ml. (You will be adding approximately 1:1 volume ratios in the following steps)
10. Solvent Extraction #1: Add an equal volume (compared to sample) of 24:1 chloroform:isoamyl alcohol to each tube.

    Note: We attempted to remove solvent extraction #1 and #2, but doing so lowers the quality and quantity of total RNA in evening primrose (*Oenothera*).
11. Vortex the tubes for 15 seconds or until the phases mix and appear cloudy.
12. Spin the tubes at maximum speed (>14,000 *g*) for 3 min in a microcentrifuge. Most of the chlorophyll will be dissolved in the lower, organic phase.
13. Pipette the upper, aqueous phase to a new 2 ml tube. Avoid transferring any of the material (usually a white precipitate) from the boundary between the phases.
14. Solvent Extraction #2: Repeat steps 10 to 13 one more time and proceed to step 15
15. Solvent Extraction #3: Add equal volume (compared to the sample) of 5:1 phenol:chlorofom (pH4.5) to the tubes containing the aqueous phase.
16. Vortex each tube for 15 seconds or until the phases mix and appear cloudy.
17. Spin the tube at maximum speed (>14,000 *g*) for 3 min in a microcentrifuge.

18. Pipette the upper aqueous phase to a new 2 ml tube, using a disposable pipette. Avoid transferring any of the material (usually a white precipitate) from the boundary between the phases.
19. Repeat steps 15 to 18 until the interphase is clean (2-3x for most *Oenothera*) and then proceed to step 20.

    Note: This is likely the most critical step. Do not proceed to 20 until the interphase appears clean.
20. Solvent Extraction #4: Add an equal volume (compared to sample) of 24:1 chloroform:isoamyl alcohol to each tube containing the aqueous phase.
21. Vortex each tube for 15 seconds or until the phases mix and appears cloudy.
22. Spin the tubes at maximum speed (>14,000 *g*) for 3 min in a microcentrifuge.
23. Pipette the upper, aqueous phase to a new 2 ml tube. Avoid transferring any of the material (usually a white precipitate) from the boundary between the phases. At this point the interphase should be relatively clean, especially if you repeated the phenol/chloroform steps to the point where the interphase appeared to be clean.
24. Repeat steps 20 to 23 one more time and then proceed to step 25.
25. Estimate the volume of the aqueous phase based on the markings on the tube. Add at least 0.5 volumes of buffer RLT and mix well by briefly shaking.

    Note: We attempted excluding this step but doing so decreased the RNA yield and quality)
26. Estimate the new total volume in the tube, and add 0.5 volumes of 95-100% ethanol. Mix by briefly shaking. If you hold the tube against the light while mixing, you sometimes see the RNA swirl while it precipitates out.
27. Pipette the contents of the tube into a Qiagen miniRNA spin column (pink), until the column is almost filled with liquid (≤750µl).
28. Cap the tube and spin for 15 seconds at >8,000 *g*. The column should be empty at the end of this spin.
29. Discard the flow-through from the collection tube.
30. Repeat the previous two steps with the same spin column, until all of the liquid in all the tube(s) from the same sample have passed through the column. The nucleic acid is now bound to the silica membrane in the spin column.
31. Apply 350µl of buffer RW1 to the spin column.
32. Cap the tube and spin for 15 seconds at >8,000 *g*. The column should be empty at the end of this spin.

33. Discard the flow-through from the collection tube.
34. Apply 80µl of DNase digestion solution to the membrane of the spin column.
35. Incubate at room temperature for 15 min.
36. Apply 350µl of solution RW1 to the spin column.
37. Cap the tube and spin for 15 seconds at >6,300 *g*. The column should be empty at the end of this spin.
38. Discard the flow-through from the collection tube.
39. Apply 500µl of solution RPE to the spin column.
40. Cap the tube and spin for 15 seconds at >6,300 *g*. The column should be empty at the end of this spin.
41. Discard the flow-through from the collection tube.
42. Apply 500µl of solution RPE to the spin column.
43. Cap the tube and spin for 15 seconds at >6,300 *g*. The column should be empty at the end of this spin.
44. Discard the flow-through from the collection tube.
45. Transfer the spin column to a new collection tube, and spin at maximum speed for 3 min. to remove remaining liquid from the silica membrane.
46. Transfer the spin column to a new 1.5 ml conical bottom microcentrifuge tube
47. Add 44µl of RNase-free water onto the column. Incubate for 1 min to eluteat room temperature and spin to to elute.

Protocol 10: TRIzol LS Reagent Method

Implemented by: Michael Melkonian and Barbara Surek (algae) and Juan Carlos Villarreal (bryophytes)

This protocol follows the procedures provided with the TRIzol LS Reagent (Invitrogen). TRIzol LS Reagent is a monophasic solution of phenol and guanidine isothiocyanate that can be used in isolation of total RNA from a wide variety of tissues and organisms, in addition to plants. This protocol was used in the isolation of total RNA from some algae samples (see Supplementary Table 1).

<u>Reagents</u>

- Chloroform or BCP (1-bromo-3-chloropropane)
- Isopropanol
- 75% Ethanol (in DEPC-treated water)
- Potassium acetate

- TRIzol LS Reagent (Invitrogen)
- RNase-free water

<u>Procedure</u>

Note: All centrifugation steps are performed at 4°C.

1. Centrifuge at lowest speed to cause algae to form pellet and wash several times with sterile culture medium (not DEPC-treated); after washing, the algal material is aliquoted into portions of 250 µl (ca. 50-100 mg packed cell volume)

2. Homogenize each 250 µl portion of pellet material to a powder in liquid nitrogen using mortar and pestle prechilled with liquid nitrogen

3. Add 750 µl TRIzol LS to each 250 µl of homogenized algal material; add more nitrogen if needed (see also Procedure described in Protocol 9)

4. Homogenization is continued until the TRIzol is pulverized as well

5. Thaw and aliquot homogenate into several Eppendorf tubes

6. Add 50 µl potassium acetate (0.2 M final concentration) to each sample

7. Incubate for 5 min at 20°C

8. Add 200 µl chloroform (for polysaccharide-rich algae), or 100 µl BCP to each sample and shake samples for 15 seconds

10. Incubate at 20°C for 10 min.

11. Centrifuge samples at 12,000g for 15 min.

12. RNA will remain in the upper, aqueous phase (ca. 70% of the applied TRIzol)

13. Carefully transfer each RNA phase into RNase-free 1.5 ml tubes

14. Add 500 µl isopropanol

15. Incubate for 1h at -20°C

16. Centrifuge at 12,000g for 10 min

17. Wash pellet with 75% ethanol

18. Gently suspend pellet in solution

19. Centrifuge at 7,500g for 5 min

20. Repeat ethanol wash steps

21. Dry pellet at 50°C for 5-10 min

    Note: appearance of drying pellet is important: drying should be terminated when the pellet begins to become transparent; contaminated RNA remains white)

22. Add RNAse-free water. Incubate at 55-60°C for 10 min.

23. Dissolve pellet completely by pipetting

Protocol 11: Tri Reagent Method

Implemented by: Michael Melkonian and Barbara Surek

The protocol for RNA isolation using Tri Reagent (Molecular Research Center, Cincinnati OH) is analogous to the TRIzol LS reagent protocol except that 1 ml of Tri Reagent is added to each 250 µl of homogenized material (= approx. 50-100 mg packed cell volume) (step 3, Protocol 10). For background, TRI Reagent combines phenol and guanidine thiocyanate in a monophasic solution to inhibit RNase activity during RNA isolation.

In cases of high RNA concentration (> 1000 ng/µl), but low 260/230 ratio (less than 1), RNA can be extracted and precipitated again. For this, steps 8-13 of the TRIzol LS Reagent Method are repeated. The RNA phase is mixed with 500 µl 8 M LiCl by vortexing and incubating for 1 h at 4°C. Subsequently, steps 16-23 of the TRIzol LS reagent protocol are performed.
Frequently, repetition of extraction decreases previous RNA concentrations up to tenfold, but increases the 260/230 ratio remarkably attaining values of 1.8 or more. Neither using isopropanol for the second precipitation process nor replacing isopropanol by LiCl as a main precipitator is as efficient as the method described above.

Protocol 12: Hot Acid Phenol Method for Angiosperms

Implemented by: Sarah Covshoff, Rowan Sage and Julian Hibberd

This RNA isolation method is a multi-component method involving an initial extraction by hot acid phenol and then a purification and DNase treatment using the RNeasy Mini Kit by Qiagen. The method described below is a modification of a method described by van Tunen et al.[3].

<u>Reagents</u>
Extraction Buffer :

- 100 mM Tris pH9.0
- 1% SDS (v/v, starting from 10% SDS stock solution)
- 100 mM LiCl
- 10   mM EDTA
- RNase-free water

Note: All components were filter purified and then the final reaction buffer was also filter purified using a Millipore Stericup.

Other reagents:

- Acid phenol (pH 4.3)
- Chloroform:isoamyl (24:1)
- 70% ethanol (diluted in RNase free H_20)

- 4M LiCl

<u>Procedure</u>

1. Add 3 ml of saturated acid phenol (pH 4.3) and 3 ml of RNA extraction buffer to a 15 ml snap cap tube. Warm tube to 65°C in a fume hood using a heat block.
2. Homogenize tissue in liquid nitrogen using a mortar and pestle.
3. Transfer up to 1.0 g of ground tissue to a tube containing the phenol-buffer mixture and close the snap cap tube completely (second stop). Immediately mix tube by hand, vortex until the phases mix and appear cloudy and keep at room temperature or 65°C depending on whether SDS is precipitating.

   Note: Use a spatula and funnel chilled in liquid nitrogen to transfer the powdered tissue to the tube.
4. Centrifuge at 2500 g for 10 min.
5. Transfer aqueous phase to a new 15 ml tube.
6. Add 3 ml of chloroform:isoamyl (24:1 ratio), close the snap cap tube completely (second stop) and mix contents immediately by hand. Then vortex until the phases mix and appear cloudy.
7. Centrifuge at 2500 g for 10 min.
8. Transfer aqueous phase to a new 15 ml tube.
9. Precipitate nucleic acids by gently adding 3 ml of isopropanol to the tube followed by gentle inversion to mix the phases. Precipitate for 1 hr at 4°C.
10. Centrifuge at 2500 g for 10 min.
11. Remove the supernatant without dislodging the pellet.
12. Rinse the pellet with 1 ml of chilled 70% ethanol made with RNase-free H_2O
13. Transfer pellet and ethanol to a new 1.5 ml tube and invert to wash.
14. Centrifuge at 16,000 g in a microcentrifuge for 2 min and remove the supernatant.
15. Spin dry the RNA pellet by successive removal of any remaining liquid following 1 min centrifugations at maximum speed in a microcentrifuge. Repeat this process until no liquid is seen when the tube is flicked. Allow the pellet to air dry for 1 to 5 min, depending on the size of the pellet.

    Note: Avoid over-drying since this will negatively affect re-suspension in water.
16. While on ice, re-suspend the pellet in 500 µl RNase-free H_2O using gentle pipetting.

    Note: Ensure the pellet is completely re-suspended before proceeding to step 17.
17. Add 500 µl of 4 M LiCl and precipitate the RNA overnight at 4°C.
18. Centrifuge at 16,000 g for 30 min at 4°C in a microcentrifuge.

19. Remove supernatant by pipette and wash the pellet three times with 200 µl of chilled 70% ethanol diluted in RNase-free H_2O. Centrifuge at 4°C.

    Note: Remove the pellet from the wall of the tube to wash the pellet more completely. Pellet will now dislodge easily and care must be taken when removing the supernatant between washes.

20. Spin the pellet, remove ethanol and dry as in step 15. Centrifuge at 4°C.
21. Dissolve pellet in RNase-free H_2O on ice.

    Note: The volume of the water is dependent on the size and clarity of the pellet, as well as the viscosity of the re-suspension, but 50 µl can be used as a standard starting point.

22. Centrifuge RNA extract for 1 min at 4°C and check concentration and integrity using a NanoDrop 2000 spectrophotometer.
23. Purify and DNase treat up to 100 µg of RNA on a RNeasy Mini Kit spin column (pink) by Qiagen.

    Note: Follow the ‘RNA Cleanup’ protocol as published in the RNeasy Handbook, including the on-column DNase digestion with the RNase-Free DNase Set by Qiagen.

24. Elute RNA twice from the column using 40 µl of 95°C RNase-free water to a total of 80 µl. However, if the mass of RNA applied to the column is less than 50 µg, then use only 40 µl to elute.

Protocol 13: Trizol/RNaqueous Midi-Kit

Implemented by: Megan Rolf and Toni M. Kutchan

Note: (two samples from C. dePamphilis and P. Ralph only used the RNAqueous Midi-Kit)

This protocol is based on a combination of two methods: The Trizol method described by Chomczynski and Sacchi[4] and the Ambion RNAqueous Midi-Kit (Life Technologies, Carlsbad, CA), with minor modifications.

<u>Reagents</u>

Extraction buffer:

- 0.8 M guanidine thiocyanate,
- 0.4 M ammonium thiocyanate,
- 0.1 M sodium acetate pH 5.0
- 5% glycerol
- 38% phenol (pH 4.3 -5.0)

Other reagents and equipment:

- 100 % ethanol
- 70 % ethanol

- Chloroform
- Isopropanol, 99.5% DNase, RNase, and Protease free
- Nuclease free H_2O
- RNAqueous-Midi Kit
- Turbo DNA-*free* kit
- 18 G ½ needle
- 14 ml polypropylene round-bottom tube
- 10 ml syringe
- 3 ml syringe
- 50 ml centrifuge tube

Procedure

1. Quickly weigh out 3 g of frozen tissue and place in a pre-chilled mortar containing liquid nitrogen.
2. Grind the sample into fine powder without allowing it to thaw.
3. Add the sample into a 50 ml centrifuge tube containing 30 ml of extraction buffer (10 ml buffer/g tissue).
4. Vortex for 1 min, then incubate at room temperature for 5 min.
5. Add 6 ml of chloroform (2 ml chloroform/g tissue) and shake vigorously for at least 20 seconds.
6. Centrifuge at 3095 *g* for 20 min. at 4°C.
7. Move supernatant to new 50 ml tube. Add one volume isopropanol (about 22.5 ml) to precipitate RNA.
8. Invert gently several times and then incubate at room temperature for 10 min.
9. Centrifuge at 3095 *g* for 10 min. at 4°C.
10. Wash pellet with 45 ml of 70 % ethanol and centrifuge at 3095 *g* for 2 min. at 4°C. Repeat once.
11. Air dry pellet for 5 min (if not completely dry, it's still okay to move on to the next step).

    Note: The following steps use the RNaqueous midi kit from Ambion (AM1911).
12. Dissolve the pellet in 5 ml of lysis/binding Solution.

    Note: Heating at 37 °C and vortexing will help dissolve pellet.
13. Heat 4.5 ml of elution solution to 100°C (for later use). Use a 17 X 100 mm round bottom sterile polypropylene tube with loose-fitting dual position cap.
14. Add 5 ml of 64% ethanol to RNA in lysis/binding solution and draw into a 10 ml syringe through an 18 gauge needle.
15. Remove needle and attach filter unit. Slowly push the lysate/ethanol mixture through. Note**:** Often times the filter gets clogged. There are tips in the kit manual for dealing with this but we found these did not help. We tried to get the solution through one filter

even with some intense pressure. Sometimes two filters were required and the following wash/elution steps were performed on both filters.

16. After filtering the solution, force air through using a clean 10 ml syringe until no more white foam is expelled (at least 3 or 4 times).
17. Wash with 100% volume of Wash Solution #1 (using syringe). Use a clean 18 g needle to draw up solution.
18. Force air through a few times again.
19. Wash with 70 % volume Wash Solution #2/3. Repeat once using syringe.
20. Force air through again until no more water droplets or fine spray can be seen.
21. Elute at least 2 times into a 2 ml tube (only use 500 ul at a time, so elute three times per tube to get about 1.5 ml total) using 100°C Elution Solution and a sterile 3 ml syringe.
22. LiCl precipitate each sample using the LiCl provided in the kit.
    a. Add ½ volume of LiCl.
    b. Place at -20°C for at least 30 min..
    c. Centrifuge at maximum speed in a microcentrifuge (about 16,000 *g*) for 15 min at room temperature.
    d. Wash pellet with 1 ml of 70 % ethanol.
    e. Centrifuge again with the same conditons for 5 min.
    f. Remove supernatant and air dry pellet.
    g. Resuspend in 50 or 100 µl RNase free H_2O.

    Note**:** Heating and vortexing can help with this.
23. DNase Treatment using the TURBO DNA-free kit from ambion (AM1907)
    a. Add 1.5 µl of DNase + 0.1 volumes of 10X buffer.
    b. Incubate at 37°C for 25 min.
    c. And another 1.5 µl of DNase.
    d. Incubate at 37°C for another 25 min.
    e. Add 0.1 volumes DNase Inactivation Reagent.
    f. Incubate 5 min at room temperature, flicking tubes every minute.
    g. Centrifuge at 10,000 *g* for 1.5 min.
    h. Move supernatant to a new tube.
24. LiCl precipitate the supernatant again (same as above). The final resuspension volume should be between 50-100 µl using RNase free H_2O.

Protocol 14: Ambion Trizol RNA Extraction in Microcentrifuge Tubes with Turbo DNA-free Digestion

Implemented by: Ingrid Jordon-Thaden and Nicholas Miles (Soltis Labs)

This procedure eliminates the mortar and pestle homogenization of tissues and instead grinds tissue in 2 ml microcentrifuge tubes. The method closely follows Ambion's protocols and could be used in a 96-well format. This method worked great for species that proved to be difficult to extract with other methods (i.e. woody and aquatic plants). Listed below are two slightly different methods for tissue collection: one directly in microcentrifuge tubes, and one in 50 ml tubes.

Note: both the addition of β-mercaptoethanol in extraction and high salts (recommended by Ambion: 0.8M sodium citrate and 1.2M NaCl) in precipitation were tried with this method and yield or quality was not affected. The addition of Sarkosyl significantly improved both yield and quality.

Reagents

- 20% bleach
- 95% ethanol
- RNase Zap
- liquid N_2
- Trizol Reagent (Ambion, Life Technologies, Carlsbad, CA)
- 20% Sarkosyl (optional for difficult species)
- 100% Chloroform
- 100% isopropanol
- 75% Ethanol
- Turbo DNA-free Kit (Ambion, store at -20°C)
    - 10X Turbo DNAase buffer
    - Turbo DNAase
    - DNAase Inactivation Reagent

Other materials

- Zirconia beads, prebaked at 200°C for 4 hours
- P1000 RNA-free filter tips
- P100 RNA-free filter tips
- RNAase free pipettes: P1000, P100
- Stretch winter gloves
- Nitrile gloves (to wear over winter gloves, latex will crack in liquid N_2)
- Two large plastic Eppendorf tube racks (96 well)
- Black shaker block (for automatic shaker/pulverizer machine)
- One ice bucket to hold the Eppendorf tube rack on ice
- One small ice bucket with liquid N_2 to hold the black shaker block

- One small ice bucket with liquid N_2 to hold the tubes while weighing
- 24 – 2 ml RNA-free Eppendorf tubes (to freeze the tissue in; note, do not use cheap tubes as they will crack)
- 72 – 1.7mL RNA-free tubes (3 batches of 24, pre-labeled; 96 tubes if you plan on using the Sarkosyl step, this adds one more chloroform extraction)
- 3x – 50mL Falcon tubes
    - to hold 100% chloroform
    - to hold 100% isopropanol (also called 2-propanol)
    - to hold 75% Ethanol

Equipment:

- Automatic shaker for tissue homogenization
- 24-place centrifuge cooled to 4°C
- Vortexer
- Water bath at 50°C
- Incubator at 37°C with orbital shaker

Concise version of Protocol (longer version available on request from Ingrid Jordon-Thaden, ingridejt@gmail.com)

Leaf Collection in microcentrifuge tubes (best to use 60 to 100 mg of tissue for high throughput)

Pre-label RNAase free 2mL tubes, place 5 zirconia beads (pre-baked at 200°C for 4 hours) in each tube and place in boxes. Cut leaf tissue and put into the tube, then directly into small cooler with liquid N_2. Store the boxes with the leaves in -80°C freezer.

Leaf collection in 50mL Falcon tubes (for large collections so many extractions can be made on the same sample if necessary)

Identify the species to collect, give it a collection number, and write the number of the 50 ml Falcon tube. Use a scissor or pin to cut a hole in the top of the tube. Fill the tube with N_2 and place in cooler with N_2 and rack. Clean scissors with Ethanol and RNAzap. Cut the youngest leaf tissue and immediately put into the Falcon tube for freezing. Take a specimen of the plant for a voucher. Place the tubes in the -80°C.

Procedure (note all centrifuge steps at 4°C unless otherwise specified)

1. Clean all surfaces and equipment with 20% bleach, 95% ethanol, and RNase Zap, and place supplies in hood.
2. Prepare sample tubes with labels.
3. Fill microcentrifuge tubes with 5 zirconia beads (if they have not been done before tissue collection)
4. Prepare aliquots of the following solutions into 50mL Falcon tubes

    i. 75% ethanol, 100% chloroform, 100% isopropanol

5. Fill dewar with liquid N_2.
6. Get samples from -80°C and place them in the black shaker block that is sitting in a bath of liquid N_2 (if already in microcentrifuge tubes), and check labels.
7. Prepare frozen tissue in microcentrifuge tubes from 50 ml Falcons (if tissue is not in them already)
    a. Fill two small coolers with liquid nitrogen (one for microcentrifuge tubes, the other for Falcon tubes)
    b. Doing about 3 or 4 plants at a time, use forceps or a spatula to move 60 to 100 mg of tissue into the microcentrifuge tubes from the 50 ml Falcon tubes.
    c. Get a weight for each of the samples, add more if necessary.
        i. This kit requires 60 to 100 mg of plant tissue.
        ii. Note: if you are doing aquatic plants use 2x the weight since so much of the weight is water.
    d. Repeat these steps until you have finished all of the samples.
8. Before pulverizing the frozen tissue, check each tube for beads, making sure they are easily moving within the tube.
9. Taking the black shaker block with the 24 samples, place tightly into the automatic shaker, doing this quickly as to not allow thawing. Shake for 2 min (they will stay frozen).
10. Place block back into the liquid nitrogen cooler if needed to shake another 2 min, then shake a second time after the block appears to be frozen.
    a. Keep on liquid nitrogen, until Trizol is added.
11. Add 1 ml of Trizol solution.
    a. Optional adjustment is to add 50 to 100 µl of 20% Sarkosyl to each sample with the Trizol.
    b. Before opening the tubes, tapping the tube on the bottom on the bench will empty most of the leaf tissue that is in the lid from the shaking process.
    c. If it does not move, you can use the vortexer with the solution added to force it out of the lid.
12. After each tube has had the Trizol solution added, vortex immediately, both the top and bottom of the tube, until all tissue is hydrated. Vortexing for 2 min can be common.
13. Then place tube on ice, and do this sequentially until you have all 24 tubes finished.

14. Once the batch is ready, incubate at room temperature (RT) for 5 min.
15. Centrifuge at 12,000 *g* for 10 min at 4°C
16. Pipette aqueous solution to a new 1.7 ml tube (will be 900 to 800 µl)
    a. If Sarkosyl is used, be aware it will be a thick, viscous layer at the interface. Try not to pull any into the aqueous layer.
17. Add 200 µl of 100% chloroform to each tube (do not change this volume or more protein will be forced into the aqueous layer)
18. Vortex for 10-15 sec (solution should be milky colored)
19. Incubate at RT for 10 min.
20. Centrifuge at 12,000 *g* for 15 min at 4°C (be careful not to disturb layers)
21. Remove upper aqueous layer (should be clear), 500 to 700 µl, and put in new 1.7 ml tube
22. If you suspect the sample does not look "clean" or if you had used the Sarkosyl addition, repeat steps 6c to 6g (i.e. the chloroform step).
23. Precipitate and pellet the nucleic acids by adding 500 µl of isopropanol, and mix by inverting the rack.
24. Incubate at RT for 10 min.
25. Centrifuge at 12,000 *g* for 15 min at 4°C
26. Pour off supernatant as waste.
27. Add 1000 ml of 75% ethanol to tube with pellet and vortex until the pellet is loose.
28. Centrifuge at 8900 *g* for 5 min at 4°C.
29. Pour off ethanol into beaker and tap tube on tissue to pull as much ethanol off as you can.
30. Centrifuge the "empty" tube for 2 min at 4°C
31. Using pipette, pull off excess ethanol collected at the bottom of the tube.
    a. Final pellet should be clear. If white, then may still have salts and you can repeat the ethanol wash a second time, however, the DNA removal step seems to also remove salt contamination.
32. Let the pellet dry for 2 min at room temperature, but no more than 10 min.
33. Re-dissolve in 50 µl of RNase-free water. If the RNA is pure, it should be instant.
    a. To aid in dissolution, incubate at 55°C for 10 min in water bath, vortex gently when finished.

Note: Never incubate longer than 10 min at this temperature. Also never increase the temperature, as this can cause RNA degradation. If pellet does not dissolve immediately,

store at 4°C overnight (or until dissolved). However, the best samples dissolve with no trouble.

34. Check on nanodrop for concentration. Samples should be diluted to be less than 200 ng/µl before proceeding to the DNAase steps. However, we have done the following steps with samples that are up to 700 ng/µl and had success. We suggest only diluting when you have 2000 or 3000 ng/µl of RNA.
35. Removal of DNA using Turbo DNA-free kit by adding 0.1 volume of 10X Turbo DNase buffer (usually 5 µl if no dilution of RNA was made) and add 1 µl of Turbo DNase to the RNA (always only 1 µl) and mix gently.
36. Incubate at 37°C while shaking on the orbiter inside the incubation oven for 30 min.
37. Add resuspended DNase Inactivation Reagent (typically 0.1 volume; 5 µl if no dilution of RNA was made) and mix well (vortex very briefly).
38. Incubate at RT for 2 min, vortexing occasionally.
39. Centrifuge at 10,000 *G* for 1.5 min at 4°C and transfer to a new tube.
40. Measure RNA with nanodrop again.
    a. 100 ng/µl is ideal, but there will most likely be more. Expect some loss from the Turbo kit. Also, if the first time the spectra appeared contaminated, this step may have cleaned it some. The OD 260/280 ratio should be 1.8 to 2.2 (not less than 1.6), in order to get good transcriptome library construction.

Protocol 15: Hot Acid Phenol Method for Algae

Implemented by: Falicia Goh and Neil Clarke

This RNA isolation method is modified from that described by Köhrer and Domdey[5]

<u>Reagents</u>
Extraction Buffer:

- 1% SDS (v/v, starting from 10% SDS stock solution)
- 51 mM sodium acetate pH5.5
- 10 mM EDTA
- DEPC treated water

Note: The final reaction buffer was filter purified using Nalgene 0.22µM filter.

Other reagents:

- Acid phenol (pH 4.3)
- Phenol:chloroform (5:1) acid equilibrated to pH4.7 from Sigma
- Isopropanol
- 70% ethanol (diluted in DEPC treated water H_20)

- 3M Sodium acetate pH5.5

Procedure

1. Preheat phenol and phenol:chloroform to 65°C. Heated phenol should not be re-used.
2. Collect algae cells via centrifugation for 10 min at 16100 *g* at room temperature. Flash freeze pellets with liquid nitrogen and keep at -80°C until extractions are carried out.
3. Re-suspend frozen pellet in 800µl of preheated extraction buffer.
4. Immediately add 800 µl of hot acid phenol. Vortex the tubes for 15 seconds.
5. Incubate at 65°C for 10 min. Vortex every 1 min for 10 sec.
6. Centrifuge at 16100 *g* at 4°C for 5 min.
7. The aqueous phase was transferred to fresh 1.5 ml micro-centrifuge tube.
8. Repeat steps 5 - 7. Repeat 3X (depending on the amount of cells used).
9. Extract with equal volume of phenol:chloroform (5:1). Vortex for 1 min at room temperature. Spin for 5 min in a microcentrifuge at top speed. Repeat step 9 three times.
10. Transfer aqueous phase to a new 1.5 ml microfuge tube. Volume should be ~700µl.
11. Add 1/10 volume of 3 M sodium acetate, pH 5.5, and 1 volume of isopropanol. Hold at 4°C for 30 min or more.
12. Spin in micro-centrifuge in 4°C at top speed for 20 min.
13. Remove the supernatant without dislodging the pellet.
14. Wash the pellet with 70% ethanol.
15. Invert and air dry tubes at room temperature.

The pellet was re-suspended in 50µl DEPC treated H2O. The RNAs were stored at -20°C.

Protocol 16: CTAB-Hot Acid Phenol Method for Algae

Implemented by: Falicia Goh and Neil Clarke

This RNA isolation method is a combination and modification of the hot acid phenol method (protocol 14) and that described by Asif et al[6]. This method was used for two taxa (*P. cruentum* and *B. braunii*).

Reagents

Extraction Buffer:

- 100 mM Tris-HCl pH8.2
- 1.4 M NaCl
- 2% CTAB

- 20 mM EDTA pH8.2
- 1 µl of 2-mercaptoethanol per ml of buffer just before use
- DEPC treated water

Note: The final reaction buffer was filter purified using Nalgene 0.22µM filter.

Other reagents:

- Acid phenol (pH 4.3)
- Phenol:chloroform (5:1) acid equilibrated to pH 4.7 from Sigma
- Chloroform
- Isopropanol
- 70% ethanol (diluted in DEPC treated water H_20)
- 3 M Sodium acetate pH 5.5
- 3 M Lithium chloride

<u>Procedure</u>

1. Preheat phenol and phenol:chloroform to 65°C. Heated phenol should not be re-used.
2. Collect algae cells via centrifugation for 10 min at 16100 *g* at room temperature. Flash freeze pellets with liquid nitrogen and keep at -80°C until extractions are carried out.
3. Re-suspend the frozen pellet in 800µl of preheated extraction buffer.
4. Incubate at 65°C for 1 hr. Gently vortex every 15 min.
5. Cool to room temperature and add equal volume of chloroform. Shake vigorously until 2 phases form an emulsion.
6. Collect the aqueous phase by centrifuging for 10 min in micro-centrifuge at 16100 *g* at room temperature.
7. Collect aqueous phase and re-extract with an equal volume of chloroform. Centrifuge as above
8. Collect aqueous phase and add 10M LiCl to a final concentration of 3M. Allow the RNA to precipitate at 4°C overnight.
9. Recover the RNA by centrifugation at 16100 *g* at 4°C for 20 min.
10. Dissolve pellet in DEPC treated water and extract once with hot acid phenol.
11. Extract the aqueous phase with equal volume of phenol:chloroform (5:1).
12. Vortex for 1 minute at room temperature. Spin for 5 min in a micro-centrifuge at top speed.
13. Extract the aqueous phase with equal volume of chloroform.
14. Collect aqueous phase and add 1/30 volume of 3M sodium acetate pH 5.5 and 0.1 volume of 100% ethanol. Mix well and keep on ice for 30 min. Centrifuge in cold for 25 min. A white jelly-like pellet consisting mostly of polysaccharides is obtained and discarded.

15. To the clear supernatant add 3M sodium acetate pH 5.2 to a final concentration of 0.3M and 3 volumes of 100% ethanol. Allow the RNA to precipitate at -80°C for 3 h to overnight.
16. Spin in micro-centrifuge at 4°C at top speed for 20 min.
17. Wash the pellet with 70% ethanol.
18. Invert tubes and air dry at room temperature.
19. Resuspend pellets in 50 µl of DEPC treated water.

Protocol 17: Invitrogen PureLink-Qiagen RNeasy Hybrid

Implemented by: Patrick Edger and J. Chris Pires

For a small number of samples the Invitrogen Purelink RNA Mini Kit (Cat #12183-018A) was used to isolate total RNA while the Qiagen RNeasy MinElute Cleanup kit (Cat #74204) was used to purify and concentrate total RNA. The methods followed the manufacturer's instructions and thus they are not repeated here.

Protocol 18: innuPREP Plant RNA Kit

Implemented by: Michael Melkonian and Barbara Surek

A small number of algae samples were extracted using the innuPREP Plant RNA Kit (Analytik Jena, Jena Germany) with either the PL and RL lysis buffer. The method followed the manufacturer's protocols and so they are not repeated here.

REFERENCES

1. Sambrook, J. & Russell, D.W. *Molecular Cloning: A Laboratory Manual*, 3rd ed. (Cold Spring Harbor Laboratory Press, Cold Spring Harbor, NY, 2001).
2. McKenzie, D.J., McLean, M.A., Mukerji, S. & Green, M. Improved RNA extraction from woody plants for the detection of viral pathogens by reverse transcription-polymerase chain reaction. *Plant Disease* **81**, 222-226 (1997).
3. van Tunen, A.J. *et al.* Cloning of the two chalcone flavanone isomerase genes from *Petunia hybrida*: coordinate, light-regulated and differential expression of flavonoid genes. *The EMBO Journal* **7**, 1257-1263 (1988).
4. Chomczynski, P. & Sacchi, N. Single-step method of RNA isolation by acid guanidinium thiocyanate-phenol-chloroform extraction. *Analytical Biochemistry* **163**, 156-159 (1987).
5. Kohrer, K. & Domdey, H. Preparation of high molecular weight RNA. *Methods in Enzymology* **194**, 398-405 (1991).
6. Asif, M.H., Dhawan, P. & Nath, P. A simple procedure for the isolation of high quality RNA from ripening banana fruit. *Plant Molecular Biology Reporter* **18**, 109-115 (2000).
